# Ferrocenyl Quinoline-Benzimidazole Hybrids: A Multistage Strategy to Combat Drug-Resistant Malaria

**DOI:** 10.1021/acs.inorgchem.5c02689

**Published:** 2025-07-31

**Authors:** Taryn M. Golding, Larnelle F. Garnie, Tayla Rabie, Janette Reader, Lyn-Marié Birkholtz, Kathryn J. Wicht, Gregory S. Smith

**Affiliations:** † Department of Chemistry, 37716 University of Cape Town, Rondebosch, Cape Town 7701, South Africa; ‡ Department of Biochemistry, Genetics and Microbiology, Institute for Sustainable Malaria Control, 56410 University of Pretoria, Hatfield 0028, South Africa; § Department of Biochemistry, Stellenbosch University, Matieland, Stellenbosch 7602, South Africa; ∥ Holistic Drug Discovery and Development (H3D) Centre, University of Cape Town, Rondebosch, Cape Town 7701, South Africa; ⊥ South African Medical Research Council Drug Discovery and Development Research Unit, Institute of Infectious Disease and Molecular Medicine, University of Cape Town, Rondebosch, Cape Town 7701, South Africa

## Abstract

Molecular hybridization and metal incorporation are widely employed strategies for drug development aimed at enhancing pharmacological efficacy while mitigating the emergence of drug resistance. The effectiveness of these approaches is supported by numerous studies demonstrating their success against a range of diseases. Despite the deployment of malaria vaccines, effective treatment remains hindered by the persistent emergence of drug-resistant strains, contributing to an alarming global disease burden. Inspired by the antimalarial candidate ferroquine, this study focused on the design and synthesis of ferrocenyl-based quinoline-benzimidazole molecular hybrids. The hybrids were evaluated for their in vitro blood-stage antiplasmodial activity against drug-sensitive NF54 and multidrug-resistant K1 strains, exhibiting potent submicromolar activity. Notably, incorporating an *N*,*N*-dimethylaminomethyl side chain significantly enhanced activity against both strains. Further assays revealed a compound with multistage antiplasmodial activity, targeting both immature and mature gametocytes. Mechanistic studies implicated the inhibition of hemozoin formation as a key mode of action, supported by in vitro cellular heme fractionation analysis. Additionally, fluorescence assays indicated the generation of reactive oxygen species under oxidative conditions, suggesting a complementary pathway contributing to the compounds’ antiplasmodial activity. These findings highlight the potential of ferrocenyl-based molecular hybrids as promising candidates in antiplasmodial drug development.

## Graphical abstract

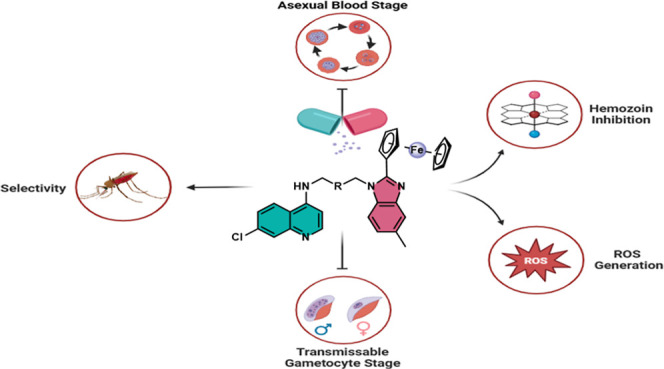

## Introduction

Malaria, a mosquito-borne infectious disease, is one of the most devastating and life-threatening parasitic diseases. Caused by the blood parasite of the genus , is the most virulent of the six protozoan species that affect humans and is largely responsible for the high levels of morbidity and mortality observed in both adults and children.^1–3^ According to the 2024 World Health Organization (WHO) World Malaria Report, the global tally of malaria cases drastically increased from 236 million in 2019 to 263 million in 2023. The additional 27 million cases have been attributed to disruptions during the COVID-19 pandemic, which has put further strain on populations within malaria-endemic countries.^1^ Despite recommendations from the WHO that include preventative measures, the rollout of the malaria vaccines RTS,S/AS01 and R21/Matrix-M, and treatment regimens such as artemisinin-based combination therapy (ACT), the prevention and cure of infections remain hindered by the acquired resistance of emerging strains to clinically used antiplasmodial drugs.^1^ Furthermore, despite reducing malaria-related deaths in vulnerable populations, such as young children, and substantially lowering the chances of severe illness, these recommended vaccines do not offer complete protection, with the highest reported efficacy being 75%.^4^ Consequently, drug discovery within this field has become increasingly challenging, leading to increased efforts to develop new strategies to both treat and prevent this devastating disease.^5–10^ This includes identifying and optimizing antiplasmodial compounds, with novel mechanisms of action, that can target multiple stages of the parasite’s life cycle within the human host.

The life cycle of the parasite consists of three main stages, namely, the liver, the asexual blood, and the transmissible gametocyte stages. Following inoculation, sporozoites travel in the bloodstream to the host’s liver, where they invade hepatocytes. Continued replication and development lead to the eventual rupturing of the hepatocytes and release of merozoites, which then invade and proliferate within the erythrocytes of the human host, initiating the asexual blood stage (ABS) of the life cycle. The sexual reproductive phase occurs within the vector; however, the switch to sexual development occurs within the vertebrate host.^11^ Here, sexually committed parasites in the ring stage develop into male and female gametocytes, a prerequisite for disease transmission.^11,12^ These gametocytes are formed from only a small proportion of ABS parasites and develop through at least five morphological stages (stages I–IV).^13–15^ An uninfected mosquito can then ingest the mature gametocytes (stage V) during its next blood meal, which is a key mediator in the transmission of the disease.^11,14–19^

The continued prevalence of this disease, coupled with the challenges of effective treatment, has prompted researchers to explore strategies to reduce transmission. Given their crucial role in the parasite’s life cycle, non-replicating gametocytes have emerged as a key target in antiplasmodial drug development.^13,20–23^ Despite many antiplasmodial agents displaying potent activity against ABS parasites, few affect the transmissible gametocyte stages.^24^ Consequently, the gametocytes remain in the human host even after clearing the ABS parasites.^25^ The ideal antiplasmodial should kill the parasite at various stages of its life cycle, such as the intraerythrocytic asexual and transmissible gametocyte stages. This multistage strategy will not only cure the patient but also offer protection for the population by preventing further transmission.^20,21^

The quinoline scaffold is a key structural component in therapeutically significant molecules and has proven efficacious in antiplasmodial chemotherapy.^16,26,27^ Indeed, the most historically significant antimalarials, chloroquine (CQ) and quinine, as well as almost all the current ACT partner drugs, incorporate the quinoline moiety. Furthermore, this pharmacophore is often the foundation of many structure–activity relationship (SAR) studies, continuing to serve as a template for the development of new chemical entities with improved activity and resistance profiles, due to its ease of chemical modifications and cost-effective synthesis.^27–30^ Their antiplasmodial activity is largely attributed to the inhibition of hemozoin (Hz) formation (vide infra), a validated target in the species. CQ and quinine, which display activity against the ABS parasites, have also been shown to kill immature gametocytes, with no effect on the mature gametocyte stages.^11,24,31^ The benzimidazole moiety is another pharmacophore with significant biological potential, particularly in the context of antiplasmodial compounds.^25,32–35^ Several benzimidazole-based compounds have been investigated for potential multistage activity, with several reported to display activity against the ABS, via the inhibition of Hz formation, and the gametocyte stages of the life cycle.^25,32,36,37^ Furthermore, the pharmacologically privileged quinoline and benzimidazole scaffolds are considered essential components of the Medicine for Malaria Venture (MMV) toolbox. These scaffolds are not only valued for their therapeutic potential but also, in some cases, as chemical probes to aid in the elucidation of mechanisms of action and target identification.^38–41^ However, these scaffolds alone may have limitations in the context of optimal potency, selectivity for over mammalian cells, and the generation of resistance.^33^

Concomitantly, researchers have explored the concept of molecular hybridization, where two different pharmacophoric groups are combined into a single molecule.^30,42–44^ The objective is to design a compound that displays activity surpassing that of the individual components, either using the same mechanisms of action as the parent compounds or by engaging additional mechanisms. This approach also offers the potential to bypass resistance mechanisms through these structurally distinct molecules, especially if the hybrid compound acts on multiple stages of the parasite life cycle. Indeed, several studies have demonstrated this approach, whereby selected pharmacophores are combined to synthesize hybrid compounds showing dual activity and/or synergistic effects (Figure 1).^45–50^ Recombining known pharmacophores could provide new leads as alternatives to the currently used antimalarials.

**Figure 1:**
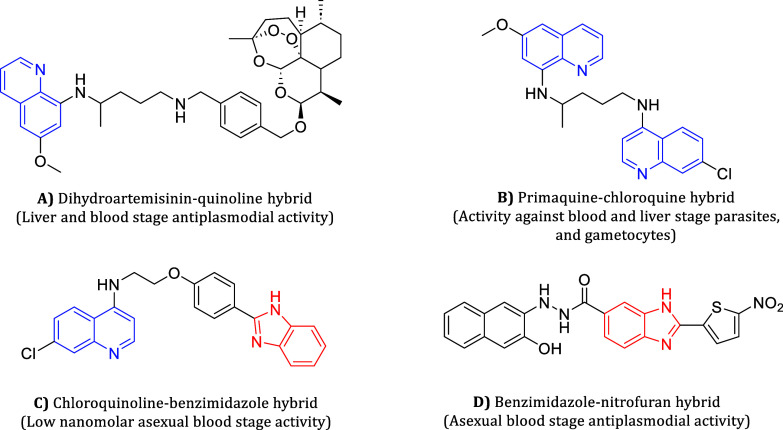
Representative examples of quinoline (blue)- and benzimidazole (red)-based hybrids (**A**,^46^ **B**,^47^ **C**,^48^ and **D** ^49^) reported to have antiplasmodial activity.

Metallofragments have also been extrapolated into the medicinal chemistry space as 3D scaffolds for fragment-based inorganic drug discovery.^51–53^ Since the discovery of the metalloantimalarial ferroquine, FQ (SSR97193), which is currently in patient-exploratory phase clinical trials, where it has demonstrated promising results,^54–58^ several studies have explored the antiplasmodial effects of incorporating ferrocene into known pharmacophoric scaffolds or existing drugs.^59–64^ Due to its physicochemical properties, such as increased lipophilicity and reduced basicity relative to CQ, FQ has been shown to selectively target and accumulate inside the parasitic digestive vacuole (DV), where it is a more potent inhibitor of Hemozoin (Hz) formation than CQ.^65^ A paper by Egan and co-workers provides a more in-depth discussion on the relationship between these physicochemical properties and the antiplasmodial activity of FQ.^66^ In CQ-resistant strains, the sustained accumulation of FQ in the DV is attributed to its differential recognition by the parasite’s transport proteins.^67^ The multiple mutations in the *P. falciparum* chloroquine resistance transporter (*Pf*CRT) protein enhance the efflux of CQ from the acidic DV into the cytosol of the parasite. FQ, however, is not recognized and transported out of the DV by the *Pf*CRT protein or other proteins involved in quinoline resistance, leading to its continued accumulation in the DV.^65^ Furthermore, the redox properties of the ferrocene/ferrocenium system of FQ can induce, directly or indirectly, additional oxidative stress in the parasite, disrupting the intracellular redox balance.^56,65^ This imbalance can overwhelm the parasites’ antioxidant defense system, resulting in a breakdown of the DV membrane and subsequent parasitic death.^56,68,69^ The ability of this compound to exhibit distinct modes of action offers the potential to mitigate the emergence of resistance. Consequently, ferrocene–pharmacophore conjugates are of interest as new antiplasmodial drug leads,^64^ with desirable properties such as metabolic stability, increased lipophilicity (potentially leading to increased absorption and efficacy), redox activity (potential to induce oxidative stress/damage), and generally low toxicity.^56,70^

Both metal incorporation and molecular hybridization have thus been synergistically investigated as methods to not only enhance the activity of potential antiplasmodial agents but to combat the emerging drug resistance of the parasite. For example, a recent study by Baartzes et al. focused on the synthesis and antiplasmodial activity of a series of ferrocenyl aminoquinoline-benzimidazole molecular hybrids, an area that has not been exhaustively explored.^71^ The synthesized ferrocenyl-hybrids were noncytotoxic against Chinese hamster ovarian (CHO) cells (CC_50_ > 50 μM), displaying excellent selectivity toward the parasite.^71^ The compounds also showed increased activity in the resistant strain, with RI values less than one (RI = 0.118–0.860).^71^ In vivo studies against the rodent mouse model, using the most active ferrocenyl compound (*Pf*IC_50_ = 0.329 μM (NF54) and 0.283 μM (K1)), which is **C1** in this study, showed a 92% reduction in parasitemia, although no mice were cured.^71^ Building on the promising results of the previous study, the current work focused on optimizing the structure of the most potent ferrocenyl compound, through structural modifications (shown by R, R′ in Figure 2) that offer increased potential for intramolecular hydrogen bonding, a feature that is reported to be crucial for the activity of FQ, and pH trapping pH trapping and the activity of FQ.^65,68^

**Figure 2:**
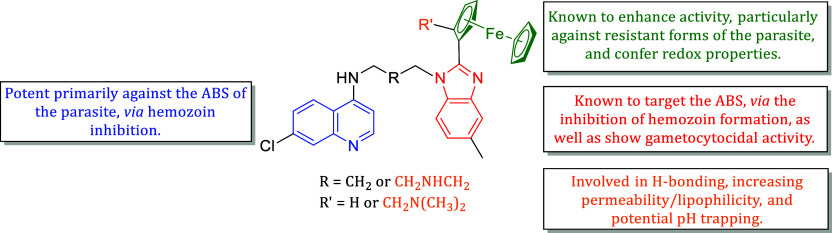
The general structure of the compounds synthesized in this study highlighting the importance of the various components.

Herein, we describe a series of ferrocenyl quinoline–benzimidazole molecular hybrids (**C1**–**C5**), evaluated for their in vitro antiplasmodial activity against the ABS and transmissible gametocyte stages of the parasite. The strategy of covalently linking two distinct pharmacologically potent scaffolds, each known to target the malaria parasite at different stages of its life cycle, and the subsequent introduction of the ferrocenyl core enables a multipronged approach, which is crucial for the successful treatment and eradication of this parasitic disease. Additionally, β-hematin (synthetic Hz) formation inhibition and subsequent cellular heme fractionation studies were conducted to provide insight into a potential mechanism of antiplasmodial action. Finally, the possible generation of reactive oxygen species (ROS) by these compounds was examined using a fluorescent probe.

## Results and Discussion

### Synthesis and Characterization

The synthetic procedure for the desired complexes started with the synthesis of precursors **1** and **2** via a nucleophilic aromatic substitution (S_N_Ar) of 4,7-dichloroquinoline with either 1,3-diaminopropane or diethylenetriamine, following a modified literature procedure.^72^ The precursors (**1** or **2**) were then reacted with 4-fluoro-3-nitrotoluene, via a S_N_Ar, to yield the corresponding nitro-containing compounds, **3** and **4**. Subsequent reduction of the nitro group to a primary amine, using ammonium chloride/zinc, yielded the amino-precursors **5** and **6** (Scheme 1). The final step to obtaining the desired ferrocenyl aminoquinoline-benzimidazole molecular hybrids, **C1**–**C4**, involved the cyclo-condensation of either precursor **5** or **6** with the appropriate ferrocenyl precursor (ferrocenecarboxaldehyde or 2-(*N*,*N*-dimethylaminomethyl)­ferrocenecarboxaldehyde), in the presence of a catalytic amount of trifluoroacetic acid (TFA). The final complexes were obtained in relatively low to moderate yields. All the precursor compounds (**1**–**6**) and complexes (**C1**–**C4**) were characterized using ^1^H (Figures S1, S2, and S9–S12) and ^13^C­{^1^H} (Figures S3, S4, and S14–S17) NMR spectroscopy as well as IR spectroscopy, and their purity ascertained using either high-performance liquid chromatography (HPLC) and/or liquid chromatography–mass spectrometry (LC–MS) (Figures S5–S8 and S18–S24). Successful synthesis of the complexes was further supported by high resolution-mass spectrometry (HR-MS) (Figures S26–S29).

**Scheme 1:**
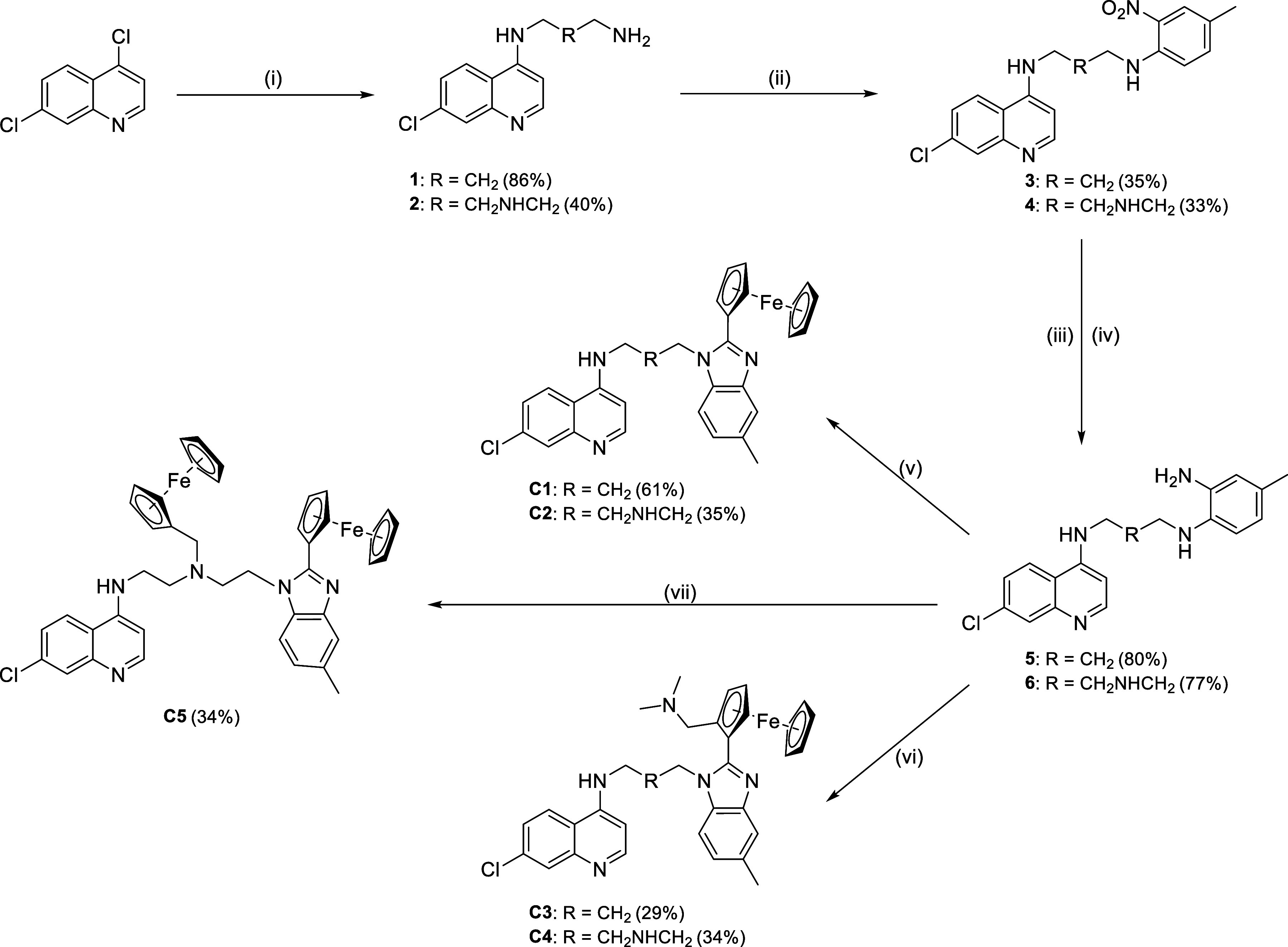
Synthetic Route Toward the Formation of Precursors, **1**–**6**, Ferrocenyl Quinoline–Benzimidazole Hybrid Complexes, **C1**–**C4**, and the Bis-Ferrocenyl Quinoline–Benzimidazole Hybrid Complex, **C5** ^a^

a Reagents and conditions: (i) 1,3-diaminopropane or diethylenetriamine, neat, 80 °C, followed by 135 °C, 22 h (**1**) or 5 h (**2**); (ii) 4-fluoro-3-nitrotoluene, anhydrous DMF, 25 °C, 24 h; (iii) MeOH, NH_4_Cl, 25 °C, 30 min; (iv) Zn dust, 25 °C, 24 h; (v) ferrocenecarboxaldehyde, TFA, MgSO_4_, EtOH, 75 °C, 2 days; (vi) 2-(*N*,*N*-dimethylaminomethyl)­ferrocenecarboxaldehyde, TFA, MgSO_4_, EtOH, 75 °C, 2 days; (vii) ferrocenecarboxaldehyde (1.5 equiv), TFA, MgSO_4_, EtOH, 75 °C, 72 h.

The reaction of precursor **6** with an excess of ferrocenecarboxaldehyde (1.5 equiv) resulted in the formation of a bis-ferrocenyl hybrid complex, **C5** (Scheme 1). The ferrocenecarboxaldehyde reacted at the diamine on the substituted phenyl ring, undergoing a cyclo-condensation reaction to form the benzimidazole, as well as at the secondary amine in the linker forming a branched tertiary amine. This complex was characterized using ^1^H (Figure S13) and ^13^C­{^1^H} (Figure S17) NMR spectroscopy, HR-MS (Figure S30), single crystal X-ray diffraction (Figure 3), and HPLC analysis (Figure S25).

**Figure 3:**
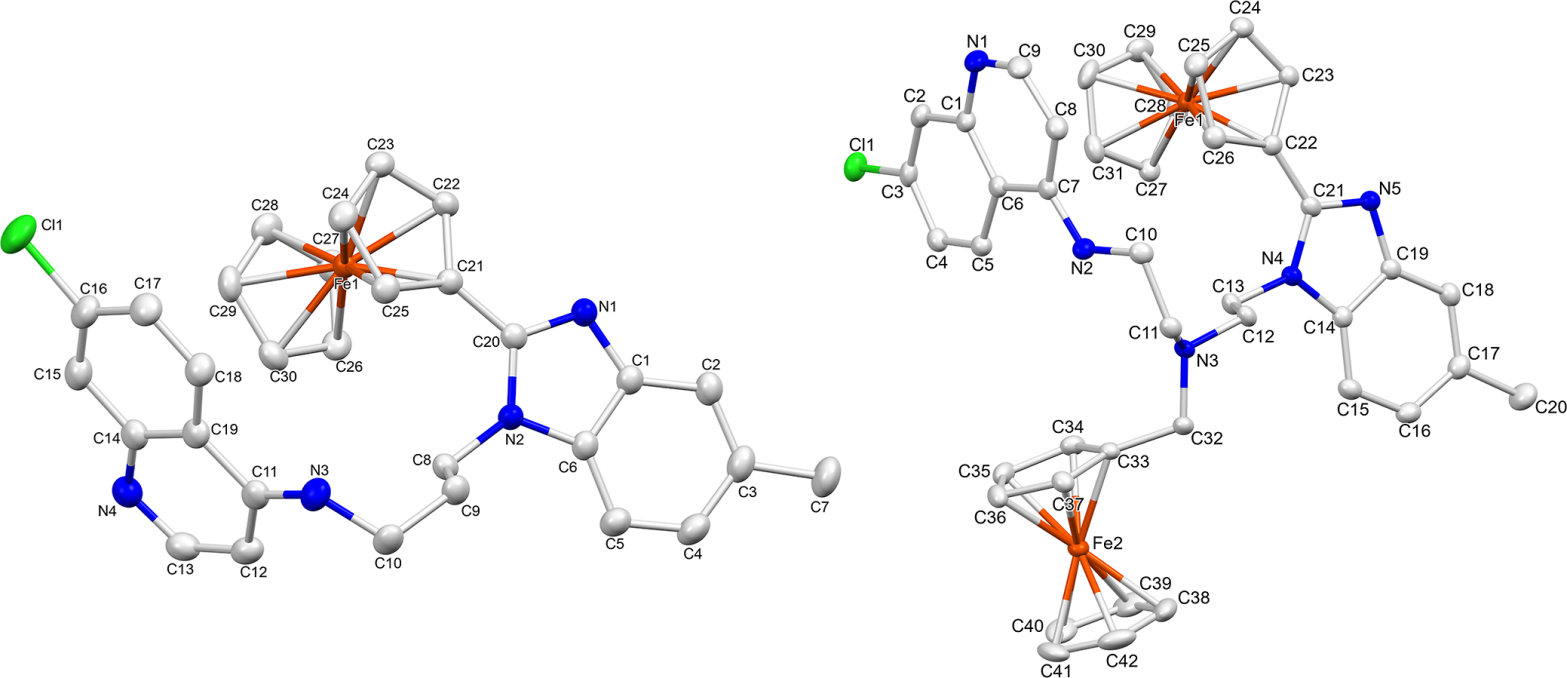
Molecular structure of ferrocenyl hybrid complexes **C1** and **C5**, with hydrogen atoms and solvent molecules omitted for clarity. Ellipsoids are shown at 40% and 50% probability levels, respectively.

Suitable crystals of the nitro-containing precursor compound **4** were obtained by the slow evaporation of a saturated methanolic solution and the molecular structure elucidated by single-crystal X-ray diffraction (Figure S31). The precursor compound crystallized in a *P*-1 space group, in a triclinic crystal system, with further crystallographic data and refinement parameters summarized in Table S1. The molecular structure of precursor **4** confirms both the successful substitution of the chloro-group at the 4-position of 4,7-dichloroquinoline with diethylenetriamine and the successful reaction of precursor **2** with 4-fluoro-3-nitrotoluene. Suitable crystals of complexes **C1** and **C5** were grown by the slow evaporation of a saturated methanolic solution (**C1**) or the slow diffusion of diethyl ether/petroleum ether into a saturated dichloromethane solution (**C5**). Complex **C1** was shown to crystallize in a *P*-1 space group, in a triclinic system, while complex **C5** crystallized in a monoclinic crystal system, in a *P*2_1_/*c* space group. In both cases, the benzimidazole and cyclopentadienyl (Cp) ring (at the 2-position of the benzimidazole) are not coplanar, with torsion angles of 27.1° (**C1**) and 11.9° (**C5**) between the two ring systems. The 3D molecular structures of **C1** and **C5** are shown in Figure 3, with further crystallographic data and refinement parameters summarized in Tables S2–S5. The ORTEP diagram of **C1** (Figure 3) confirmed the successful cyclo-condensation of the amino-precursor (**5**) with ferrocenecarboxaldehyde. Furthermore, the presence of two ferrocenyl groups in the bis-ferrocenyl complex **C5** was confirmed, as suggested by the ^1^H NMR spectrum (Figure S13) where a doubling up of the ferrocenyl signals was observed. Analysis of the bond lengths around the tertiary amine revealed carbon–nitrogen (C_11_–N_3_, C_12_–N_3_, C_32_–N_3_) bonds between 1.461 and 1.474 Å, which is in the expected range for carbon–nitrogen single bonds in similar compounds.^73–75^

It has been proposed that the oxidation of ferrocene (Fe­(C_5_H_5_)_2_) to the ferrocenium cation (Fe­(C_5_H_5_)_2_^+^) is a reversible one-electron transfer process. Under oxidizing conditions, mimicking the parasite’s DV, FQ also shows a reversible one-electron redox reaction, forming ferriquinium and generating lethal hydroxyl radicals via a Fenton-like reaction.^56,65,68,69^ Due to its high reactivity, this small amount is sufficient to induce significant damage to the DV membrane and subsequent death.^69^ This prompted the investigation into the electrochemical properties of the Fe­(C_5_H_5_)_2_/Fe­(C_5_H_5_)_2_^+^ couple in complexes **C1**–**C5**, using cyclic voltammetry. The solutions were prepared in DCM, using a scan rate of 100 mV/s, with the voltammograms shown in Figure S32. Additionally, the cyclic voltametric parameters for the solutions of the complexes and FQ are summarized in Table S6. FQ was used as the reference, as it is considered the benchmark for all newly developed ferrocenyl-based antiplasmodials.

The Fe­(C_5_H_5_)_2_/Fe­(C_5_H_5_)_2_^+^ couple in FQ and complexes **C1**, **C2**, and **C5** were found to be chemically and electrochemically reversible in the DCM/0.1 M TBAP system, with E_1/2_ values ranging between 0.537 and 0.584 V (Table S6). Despite the presence of two ferrocenyl moieties, complex **C5** undergoes a single-stepped oxidation event. The slightly larger Δ*E* value obtained for complex **C5**, compared to complexes **C1** and **C2** (Table S6), suggests an overlap of the two oxidation events and thus limited electronic communication between the two ferrocenyl units.^76–78^ The Fe­(C_5_H_5_)_2_/Fe­(C_5_H_5_)_2_^+^ couple in complexes **C3** and **C4**, however, were found to be chemically and electrochemically irreversible, as no reduction peak was observed. The irreversibility of these complexes is an interesting observation as FQ also contains a 1,2-disubstituted ferrocenyl system. However, the combined presence of the electron-withdrawing benzimidazole moiety and *N*,*N*-dimethylaminomethyl side chain are shown to affect the reversibility of this Fe­(C_5_H_5_)_2_/Fe­(C_5_H_5_)_2_^+^ couple. Overall, the reductive ability of the ferrocenyl complexes (**C1**–**C5**) suggests that these compounds may act via a Fenton-like reaction and produce hydroxyl radicals in the presence of H_2_O_2_.

### In Vitro Erythrocytic Antiplasmodial Activity and Selectivity

The quinoline–benzimidazole ferrocenyl hybrid complexes (**C1**–**C5**) were evaluated for their in vitro ABS antiplasmodial activity against both drug-sensitive *Pf*NF54 and multidrug-resistant (MDR) *Pf*K1 strains, using a lactate dehydrogenase (*p*LDH) assay.^79^ The IC_50_ values and resistance indices are listed in Table 1. Both compounds **C1** and **C5** showed low micromolar potency against the *Pf*NF54 strain. The incorporation of a tertiary amine and the additional ferrocenyl moiety in the linker of **C5** enhanced its activity over 6-fold, compared to **C1**. Complexes **C2**–**C4**, however, notably displayed submicromolar activity, with IC_50_ values ranging between 0.025 and 0.038 μM. The incorporation of the secondary amine in the linker of **C2** was shown to enhance the activity over 260 times compared to that of its propyl-containing congener **C1**, while the same substitution in **C4** compared to **C3** had minimal impact on the potency. Complexes **C2** and **C4**, both of which contain the diethylenetriamine linker, displayed equipotent activity, despite the incorporation of the side chain in complex **C4**. Complex **C3** is the most potent of the synthesized complexes against the drug-sensitive NF54 strain (IC_50_ = 0.025 μM). The enhanced activity of **C3** compared to **C1** is attributed to the conjugation of the *N*,*N*-dimethylaminomethyl side chain to the 2-position of the ferrocene. This side chain is also present in the well-known antiplasmodial agent, FQ. Interestingly, FQ favors intramolecular hydrogen bonding between the 4-aminoquinoline NH and terminal tertiary amino groups. The adoption of such a folded conformation has been reported to enhance its passage through the membrane via increased permeability, as the open conformation is expected to be less lipophilic.^65,68^ This improved membrane transport of FQ and accumulation inside the DV, relative to CQ, is a noteworthy factor in their differing antiplasmodial activity. In addition to the increased lipophilicity of **C3**, this additional basic amine in the lateral side chain is likely to also allow for enhanced pH trapping of the compound, further increasing its accumulation inside the parasite’s DV.

**Table 1:** In vitro ABS Antiplasmodial Activity of Hybrids **C1**–**C5** Against the Drug-Sensitive *Pf*NF54 and MDR *Pf*K1 Strains and Their Cytotoxicity Against the CHO Cell Line

Cmpd	*Pf*NF54 IC_50_ ± SEM (μM)^a^	*Pf*K1 IC_50_ ± SEM (μM)^a^	CC_50_ (CHO) ± SEM (μM)^b^	RI^c^	SI^d^
**C1**	9.91 ± 0.71	6.29 ± 0.51	43.7 ± 2.5	0.63	4.4
**C2**	0.038 ± 0.002	0.0094 ± 0.0009	>50	0.25	>1315
**C3**	0.025 ± 0.001	0.113 ± 0.005	6.1 ± 1.2	4.4	244.4
**C4**	0.038 ± 0.003	0.17 ± 0.02	29.8 ± 0.5	4.5	784.2
**C5**	1.51 ± 0.14	14.47 ± 1.14	>50	9.6	>33.1
CQ	0.015 ± 0.001	0.17 ± 0.01	ND^f^	11.3	ND^f^
FQ	0.020 ± 0.003^e^	0.0239 ± 0.0002^e^	ND^f^	1.20	ND^f^

a Results are the mean ± SEM obtained from samples screened in technical duplicate, over three or four biological replicates.

b Results are the mean ± SEM obtained from samples screened in technical triplicate on a single occasion.

c (IC_50_ K1/IC_50_ NF54).

d (CC_50_ CHO/IC_50_ NF54).

e Results from ref 80.

f ND = not determined

Of the compounds tested against the MDR K1 strain, complexes **C2** and **C3** are once again the most active, with submicromolar activity (IC_50_ = 0.0094 and 0.11 μM, respectively). Notably, complex **C2** is significantly (*p* < 0.01) more potent than the clinically used drug, CQ, against this resistant strain. As observed in the NF54 strain, **C1** and **C3**, which are analogous and only differ in the presence of the *N*,*N*-dimethylaminomethyl side chain, display a notable 57-fold difference in activity. Furthermore, introducing the additional amine in the linker was once again shown to result in a remarkable increase in activity (IC_50_ = 6.29 μM (**C1**) vs 0.0094 μM (**C2**)). Overall, the presence of an additional basic nitrogen atom, in either the linker or the side chain (**C1** vs **C2** and **C1** vs **C3**), has been shown to enhance the ABS antiplasmodial activity significantly (*p* ≤ 0.01). This is likely due to the nitrogen atom assisting in drug accumulation within the parasites’ DV (supported by the in vitro Hz inhibition studies, vide infra), via pH trapping, and/or possible intramolecular hydrogen bonding (vide supra).^81^ However, as observed for the NF54 strain, further introduction of an amine (**C2** vs **C4** and **C3** vs **C4**) has no appreciable impact on the antiplasmodial activity.

Furthermore, all the complexes display less cross-resistance between the drug-sensitive and -resistant strains compared to CQ (resistance index, RI = 11.3), although compounds **C3** and **C4**, which contain the *N*,*N*-dimethylaminomethyl side chain, have higher RI values (RI = 4.4 and 4.5, respectively) than **C1** and **C2**. Complexes **C1** and **C2** are more potent against the K1 strain (RI < 1), suggesting limited or no cross-resistance. The enhanced activity of these compounds against the resistant strain might suggest that they are not recognized and effluxed by mutant *Pf*CRT. This is similarly observed for FQ, where its potent activity against a CQR strain is proposed to be attributed to the introduction of the ferrocenyl core on the CQ scaffold.^65^ As a result of the altered side chain, the mutations in the *Pf*CRT which allow for efflux of the CQ molecule are no longer sufficient for the efflux of FQ from the DV.^65^

The mammalian Chinese hamster ovary (CHO) cell, which has been used in various studies to assess the pathogen-specific effects of compounds,^23,36,82,83^ was employed to evaluate the selectivity of the investigated compounds toward the parasite. The ferrocenyl complexes showed low cytotoxicity when tested against the CHO cell line, with relatively high selectivity indices (SI’s), except for **C1**, with an SI of 4.4 due to its less potent antiplasmodial activity. Comparatively, **C2**–**C5**, with a CC_50_ range between 6 and 50 μM, display clear selectivity toward the parasite, with complex **C2**, which also displayed the lowest RI value, shown to be at least 1315 times more selective toward parasitized red blood cells relative to mammalian cells.

### In Vitro Gametocytocidal Transmission-Blocking Potential

Due to the known multistage activity of various quinoline- and benzimidazole-based compounds^11,24,25,31,32,36,37,50,84^ and the urgent need for antiplasmodial agents that act across various stages of the parasite’s life cycle,^13,20–23^ the synthesized quinoline–benzimidazole molecular hybrids (**C1**–**C5**) were subsequently screened for their gametocytocidal activity against immature (stage II/III) and mature gametocytes (stage IV/V) of , with the maximum percentage inhibition of these complexes shown in Table 2.

**Table 2:** Maximum Inhibition Percentage at 10 μM of Complexes **C1**–**C5** Against Immature (>90% Stage II/III) and Mature Gametocytes (>95% Stage IV/V)

	Immature gametocytes (>90% stage II/III)	Mature gametocytes (>95% stage V)
compound	% Inhibition (IC_50_ ± S.E., μM)^a^	
**C1**	3%	7%
**C2**	75%	32%
**C3**	88% (2.35 ± 0.13 μM)	99% (7.27 ± 0.52 μM)
**C4**	59%	72%
**C5**	60%	32%
MMV390048	>77%	>93%
Methylene blue	>97%	100%

a Results reported after three independent biological repeats, each performed in technical triplicate.

Notably, the derivatized compounds (**C2**–**C5**) all showed higher percentage inhibition than the principal compound **C1**, which was nearly inactive at the tested concentration of 10 μM against the gametocyte stages of the parasite. Consistent with the in vitro ABS activity (Table 1), hybrid **C3** exhibited the highest observed inhibition against both gametocyte stages, with calculated IC_50_ values of 2.35 and 7.27 μM against immature and mature gametocytes, respectively (Table 2). Compound **C3**, however, appeared more potent against the ABS parasites, where submicromolar activity was observed (Table 1). This observation aligns with previous reports that compounds targeting the ABS stage often exhibit reduced efficacy against gametocytes, particularly in cases where target expression is stage-specific.^25^ For compounds that display dual activity, ABS-active compounds tend to show higher efficacy against early stage gametocytes with diminished activity against the later stages.^24,25^ Interestingly, hybrid **C4** demonstrated a higher percentage inhibition against mature gametocytes (72%) compared to immature gametocytes (59%) at 10 μM. These preliminary findings suggest that the derivatized compounds may possess multistage activity and could serve as promising dual-acting antiplasmodial candidates, pending further investigation.

### Inhibition of β-Hematin Formation (Cell-Free Study)

During the intraerythrocytic stage of the life cycle, the parasite degrades hemoglobin (Hb) found within the red blood cells of the host.^26,85,86^ This catabolic digestive process occurs in the acidic digestive vacuole of the parasite, with a byproduct of Hb digestion being the production of heme.^85,86^ To circumvent the adverse effects associated with the build-up of free heme, the parasite has developed a unique detoxification mechanism which involves converting the free heme into an inert, nontoxic, biocrystalline material known as Hz.^86^ Despite being nontoxic to the parasite, when leaked from ruptured erythrocytes Hz is toxic to humans, causing high fever in patients.^2,87^ Consequently, researchers have exploited this unique detoxification mechanism by developing antiplasmodial agents that interfere with Hz formation.^88,89^ It is also worth noting that parasite resistance to scaffolds that are known to target this pathway arises from mutations in the DV membrane transporter protein, chloroquine resistance transporter, not from changes in the heme detoxification pathway.^90^ Consequently, the Hz formation pathway is still considered a viable target within the drug development process of antiplasmodial agents.^39,91–93^

Upon entry into the DV, CQ has been shown to inhibit Hz formation by forming a dimeric complex with hematin through π*–*π interactions.^39,94,95^ FQ is also a reported β-hematin formation inhibitor,^96^ leading to the build-up of free heme and subsequent parasite death.^88^ The ability of a compound to inhibit Hz formation can be predicted using the biomimetic cell-free NP40 detergent-mediated β-hematin formation inhibition assay.^91,97^ This assay mimics the conditions in the acidic DV of the parasite and relies on the binding of pyridine to uncrystallized free heme to detect the inhibition of β-hematin formation using UV–vis spectroscopy (with absorbance measured at 405 nm).^91,97,98^ Consequently, the ability of the ferrocenyl quinoline-benzimidazole hybrids (**C1**–**C5**) to inhibit β-hematin (synthetic Hz) formation was investigated as a potential mechanism of antiplasmodial action.

Compounds **C1**–**C5** were found to possess β-hematin formation inhibitory activity relative to the known Hz formation inhibitor drug, CQ, as evidenced by the characteristic sigmoidal dose–response curves (Figure 4). Hybrids **C1**–**C4** display enhanced β-hematin inhibitory activity compared to CQ, while **C5**, which contains the additional ferrocenyl moiety in the linker chain, was the least active of the synthesized hybrids. Incorporation of the *N*,*N*-dimethylaminomethyl side chain was shown to improve the activity of **C3** (IC_50_ = 5.42 μM) relative to **C1** (IC_50_ = 7.26 μM), while hybrids **C2** and **C4**, which are over 2-fold more active than CQ, have near comparable activity, with IC_50_ values of 4.47 and 4.90 μM, respectively. These cell-free results collectively suggest that this series of hybrids may act as Hz formation inhibitors, given that all the complexes have reasonably good β-hematin formation inhibitory activity. However, further experiments involving cellular fractionation of heme species are essential to validate the intracellular mechanism of action. Additionally, the notable differences between the in vitro ABS antiplasmodial activities (Table 1) and β-hematin formation inhibition IC_50_ values can be rationalized by the various factors which govern whole-cell potency, including drug accumulation, permeability and other physicochemical properties.^99^

**Figure 4:**
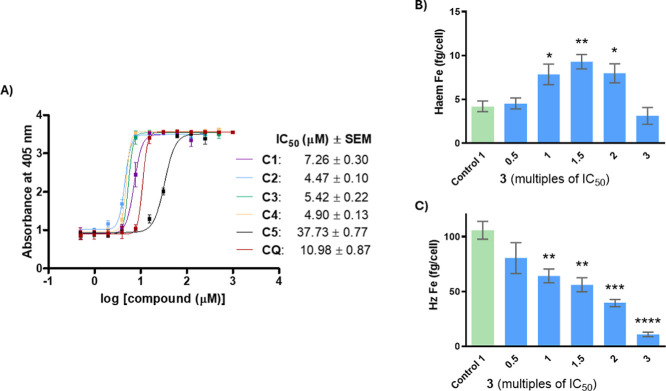
Dose–response curves and IC_50_ values obtained for the β-hematin formation inhibitory activity of complexes **C1**–**C5**, and CQ. Results are the mean ± SEM obtained from samples screened in technical duplicate, over three or four replicates (A). Heme fractionation profiles of hybrid **C3** showing the amount of “free” heme Fe (B) and hemozoin (Hz) Fe (C) in whole-cell NF54 parasites with increasing concentrations of compound **C3**. Significance was determined using the students’ two-tailed *t*-test: *p < 0.05, **p < 0.005, ***p < 0.001 ****p < 0.0001.

### Inhibition of Hemozoin Formation (In Vitro Study)

Hybrid **C3** was prioritized for further mode of action studies as it generally displayed the most potent ABS activity. The effect of hybrid **C3** on heme and Hz levels in whole-cell NF54 parasites cultured in vitro was assessed by employing the heme fractionation assay, previously described by Combrinck et al.^100^ Like the β-hematin formation assay, this assay also relies on the detection of heme species coordinated to pyridine via UV–vis spectroscopy. However, unlike the cell-free assay, the heme fractionation assay isolates both the heme and Hz components via a series of solubilization and separation steps. Furthermore, the absolute amount of each heme species per cell is calculated using the UV–vis data normalized to the flow cytometry parasite cell count. Upon exposure of the ABS NF54 parasites to increasing concentrations of compound **C3**, a statistically significant change in the levels of both heme and Hz was observed. The absolute levels of heme per cell steadily increased with a concomitant decrease in Hz levels as a function of the dosage over the range 0.5–1.5× the IC_50_ value. Interestingly, from 2× IC_50_ exposure and above, the levels of heme were observed to decrease owing to induced stress on the parasite that can lead to a lower Hb uptake.^101^ Notably though, the absolute levels of Hz per cell decreased steadily such that at 3× IC_50_, Hz levels were more than five times lower than the untreated control parasites. This suggests that exposure of the parasite to this compound, over the described IC_50_ range, inhibits Hz formation, resulting in the build-up of free heme and subsequent parasite death. These results thus corroborate the cell-free data for compound **C3**, with the cell-free activity translating to the in vitro studies, suggesting that the other derivatives would act via a similar mechanism.

### Reactive Oxygen Species Generation

During the intraerythrocytic phase of the parasites’ life cycle, the oxidative process of Hb digestion is its main source of amino acids.^65^ This degradation process generates a large amount of ferriprotoporphyrin IX and various ROS, such as hydrogen peroxide, singlet oxygen, superoxide anions, and hydroxyl radicals.^56,69^ The parasite relies on an adequate antioxidant defense system and efficient redox regulation to deal with the high flux of ROS and maintain redox regulatory processes and pathogenicity.^102^ Glutathione (GSH), for example, protects the parasite from oxidative damage, as it is an efficient trapping molecule for hydroxyl radicals.^65^ Its accumulation in the DV of ABS parasites treated with FQ is thus a response to oxidative aggression.^65^ The continued exposure of malaria parasites to high concentrations of ROS increases their vulnerability to disruptions of this redox equilibrium, offering a great opportunity to develop chemotherapeutic agents that can perturb this equilibrium.^102^

Fluorogenic redox-sensitive probes are often employed to measure the presence of ROS, due to their low cost, ease of use, and nontoxicity, allowing for spectrofluorimetric measurements.^103^ 2′,7′-Dichlorodihydrofluorescein diacetate (DCFH_2_-DA) is the most widely used fluorogenic probe.^103^ Cleavage of the acetate groups, through alkaline hydrolysis, generates 2′,7′-dichlorodihydrofluorescein (DCFH_2_), which in the presence of oxidants, yields the highly fluorescent 2′,7′-dichlorofluorescein (DCF, λ_em_ = 523 nm).^103^ The DCFH_2_ probe reacts with an array of oxidants, making it an ideal indicator of general oxidative stress. Using this approach,^103^ the hybrid complexes (**C1**–**C5**) were investigated for their ability to generate hydroxyl radicals in the presence of H_2_O_2_. The presence of these radicals was confirmed by monitoring the fluorescence over time (Figure 5). Ferrocene and FQ were used as positive controls as they are known to generate hydroxyl radicals via a Fenton-type reaction in the presence of H_2_O_2_,^68^ whereas H_2_O_2_ alone was used as a negative control. The spectra of the controls and complexes **C1**, **C3**, and **C5** are shown in the Supporting Information (Figures S33 and S34).

**Figure 5:**
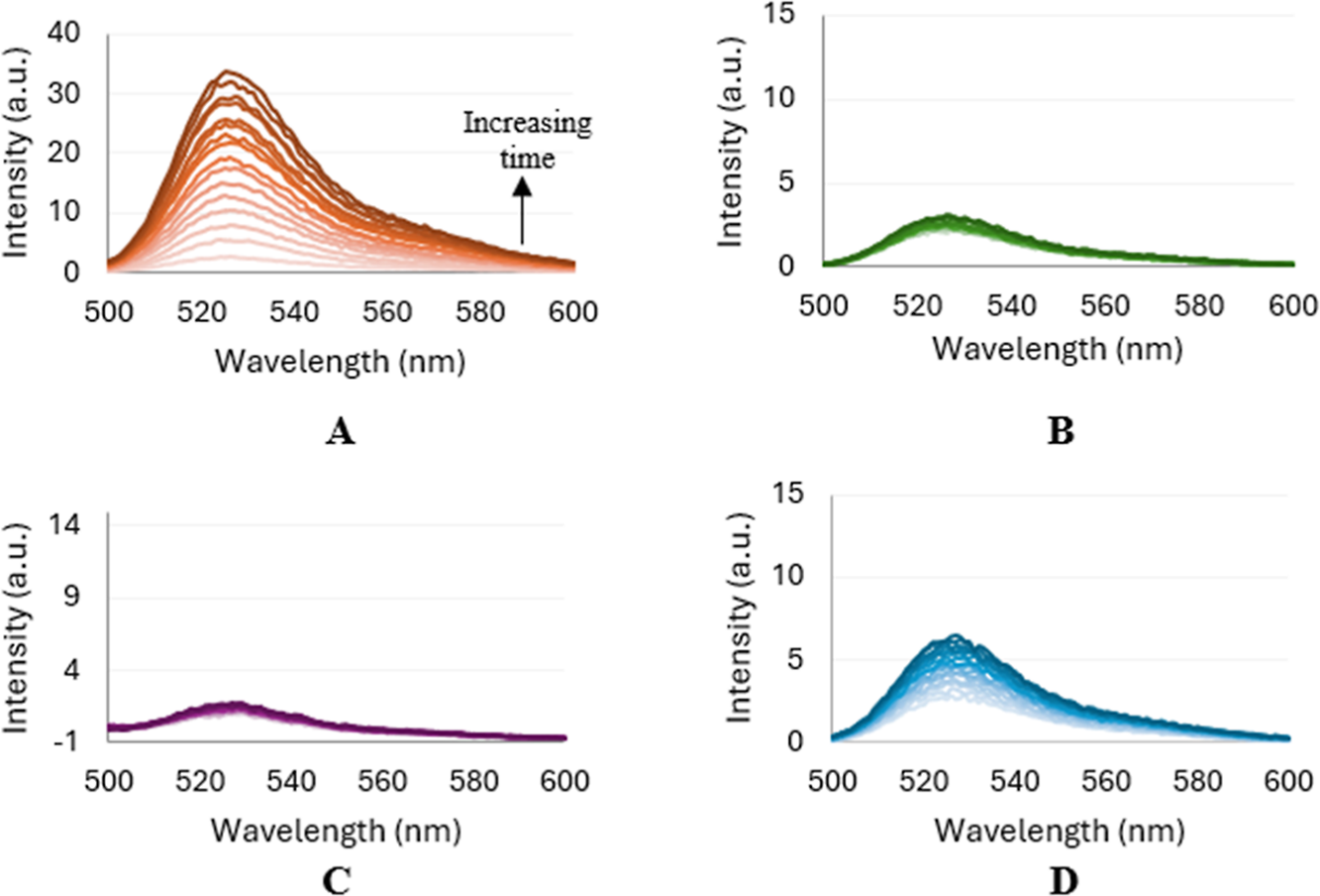
Changes in the fluorescence emission spectra of the positive control, ferrocene (A), the negative control, H_2_O_2_ (B), and hybrid complexes **C2** (C) and **C4** (D), in 1:4 DMSO/PBS, following the addition of H_2_O_2_ and the nonfluorescent fluorescein analogue, H_2_DCF, monitored over 30 min.

Hybrid complexes **C3** and **C4**, which contain the *N*,*N*-dimethylaminomethyl side chain, are shown to produce hydroxyl radicals in the presence of H_2_O_2_, as evidenced by the increasing fluorescence intensity over time (Figures S34 and 5, respectively). Despite the intensity not increasing to the same extent as observed for ferrocene (**A**), it is evident that upon comparison to the negative control (**B**), where no noteworthy change in intensity is observed over the investigated period, these two complexes display an incremental increase in intensity over time, suggesting ROS production. Although no changes in the fluorescence intensity are observed for complexes **C1**, **C2**, and **C5**, the production of ROS, as a potential mechanism of action, cannot be ruled out, as alternative ROS products may be produced, although not detectable using the given probe. It is also interesting to note that the oxidation potentials of the compounds (Table S6) appear to have no bearing on their ability to produce ROS. Ferrocene and FQ, which display the lowest oxidation potentials (*E*_pa_ = 0.543 and 0.613 V, respectively), and complexes **C3** and **C4**, which have the highest oxidation potentials (*E*_pa_ = 0.678 and 0.662 V, respectively) and display irreversible oxidation, were all shown to generate hydroxyl radicals.

## Conclusion

In summary, we have developed ferrocenyl-based quinoline–benzimidazole hybrids that selectively target ABS parasites over mammalian CHO cells and possess resistance indices less than the clinical drug, CQ, against the MDR K1 strain. Generally, the basic amine-containing complexes display nanomolar activity against the ABS of drug-sensitive and MDR parasites. In particular, complex **C2**, which contains the diethylenetriamine linker, was the most potent of the tested compounds against the MDR strain, displaying activity superior to CQ, and enhanced selectivity toward parasitized red blood cells. Investigations into the gametocytocidal action revealed that compounds **C2**–**C5** exhibit activity against both mature and immature gametocytes, with complex **C3** being the most potent, showing low micromolar activity. The therapeutic potential of complexes **C2** and **C3**, in particular, is promising and warrants further optimization. Mechanistic studies revealed that Hz formation inhibition, which remains a valid target for newly developed antiplasmodial drugs, is a likely mechanism of action for this series of complexes. Indeed, compounds **C1**–**C5** were shown to inhibit the formation of β-hematin, with in vitro heme fractionation studies, with the frontrunner **C3**, further confirming intracellular hemozoin formation inhibition as a mechanism of antiplasmodial action in the ABS. Investigations into the potential ROS-generating ability of the compounds revealed that those compounds containing the *N*,*N*-dimethylaminomethyl side chain (**C3** and **C4**) generate hydroxyl radicals under oxidizing conditions. This is a known mechanism of action for the antimalarial drug candidate FQ and is reported to be a contributing factor to its potent antiplasmodial activity. Since hemozoin formation is irrelevant in the gametocyte stages, the generation of ROS may be contributing to both the mechanism of action in gametocytes as well as in the ABS. Furthermore, there is potential for synergy between Hz formation inhibitors and ROS-generating compounds, given that both ultimately result in the generation of radicals, the mechanisms can thus potentiate each other. These findings collectively display the potential for ferrocenyl-based quinoline–benzimidazole hybrid complexes to be used as multistage antiplasmodial agents. Advantageously, they impart their activity through various mechanisms of action, with favorable selectivity, which is crucial for newly developed drugs to minimize off-target effects that could result in adverse side effects, a major issue for chemotherapeutic treatments today. Future work will involve investigations into the physicochemical and pharmacokinetic properties of the compounds, as these factors directly influence a drug’s absorption, distribution, metabolism, and excretion (ADME). Understanding these properties is essential for predicting in vivo performance and serves as a critical guide for lead optimization and preclinical development.

## Experimental Section

### Chemicals and General Methods

All reagents and solvents were purchased from commercial sources (Sigma-Aldrich, Merck, KIMIX, and Combi-Blocks) and used without further purification. All reactions were conducted under an inert argon atmosphere unless otherwise specified. Reactions were monitored by thin-layer chromatography (TLC) using aluminum-backed precoated silica-gel 60 F254 plates and compounds visualized using ultraviolet light (254 nm). Solvents were reduced using a Büchi rotary evaporator. All aqueous solutions were prepared by using deionized water. Column chromatography was conducted using 60 Å silica gel (70–230 mesh). The precursor 2-(*N*,*N*-dimethylaminomethyl)­ferrocenecarboxaldehyde was prepared according to literature.^104^

Nuclear magnetic resonance (NMR) spectra were recorded on a Bruker XR600 MHz spectrometer (^1^H at 599.95 MHz and ^13^C­{^1^H} at 151.0 MHz), a Bruker Topspin GmbH (^1^H at 400.22 MHz and ^13^C­{^1^H} at 100.65 MHz), or a Varian Mercury 300 (^1^H at 300.08 MHz) spectrometer. These were equipped with a Bruker Biospin GmbH casing and sample injector at 30 °C and tetramethylsilane (TMS) was used as the internal standard. Coupling constants are reported in Hz and chemical shifts are reported in ppm relative to residual solvent signals. Infrared (IR) spectroscopy was performed on a PerkinElmer Spectrum 100 FT-IR spectrometer, using attenuated total reflectance (ATR), with vibrations measured in units of cm^–1^. Cyclic voltammetric measurements were carried out at ambient temperature under an inert argon atmosphere using a three-electrode system on a BASi Epsilon Eclipse potentiostat/galvanostat electrochemical workstation. The C-3 cell stand, equipped with a glassy carbon working electrode, Ag/AgCl (saturated NaCl) reference electrode, and a Pt wire auxiliary electrode were used to carry out electrochemical measurements. All experiments were carried out in anhydrous dichloromethane containing 0.10 M tetra-*n*-butylammonium perchlorate (TBAP) as the supporting electrolyte.

LC–MS analysis was performed using an Agilent 1260 Infinity Binary Pump, an Agilent 1260 Infinity diode array detector (DAD), an Agilent 1290 Infinity Column Compartment, an Agilent 1260 Infinity Standard Autosampler, and an Agilent 6120 Quadrupole (Single) MS system, with an APCI/ESI multimode ionization source. The purities were determined by a Waters’ HPLC using an X-bridge C18 5 μm column (4.6 × 150 mm); organic phase: 10 mM ammonium acetate (pH 3.7) in HPLC-grade methanol, aqueous phase: 10 mM ammonium acetate (pH 3.7) in HPLC-grade water; flow rate = 1.20 mL/min; detector: PDA. The purities of all compounds were found to be >97%.

The purity of the complexes were also determined using an analytical Agilent HPLC 1260 infinity II, equipped with an Agilent DAD 1260 UV/vis detector and an Agilent Pursuit 5 C18 column (5.0 μM, 150 mm × 4.6 mm). The compounds were eluted using a mixture of solvent A (0.1% TFA in H_2_O) and solvent B (MeOH) at a flow rate of 0.5 mL/min. The gradient elution conditions were as follows: 10% solvent B between 0 and 2 min, 10–90% solvent B between 2 and 8 min, 90% solvent B between 8 and 20 min. Purity was determined at 254 nm. All compounds were confirmed to have >97% purity.

Single-crystal X-ray diffraction data were collected on either a Bruker D8 Venture diffractometer (compounds **4** and **C5**) or a Bruker KAPPA APEX II DUO diffractometer (complex **C1**), using graphite-monochromated Mo Kα radiation (χ = 0.71073 Å (**4** and **C1**) or χ = 1.54178 Å (**C5**)). Data collection was carried out at 100(2) K for compounds **4** and **C5** and 173(2) K for complex **C1**. Temperature was controlled by an Oxford Cryostream cooling system (Oxford Cryostat). Cell refinement and data reduction were performed using the program SAINT.^105^ The data were scaled and absorption correction performed using SADABS.^106^ The structure was solved by direct methods using SHELXS-97 ^106^ and refined by full-matrix least-squares methods based on *F* ^2^ using SHELXL-2014 ^106^ and using the graphics interface program X-Seed.^107,108^ The programs X-Seed and POV-Ray^109^ were used to prepare molecular graphic images.

Compound **4**: all non-hydrogen atoms were refined anisotropically. All hydrogen atoms, except the hydroxyl and amino hydrogens, were placed in idealized positions and refined in riding models with *U*_iso_ assigned 1.2 or 1.5 times *U*_eq_ of their parent atoms and the C–H bond distances were constrained from 0.95 Å to 0.99 Å. The other hydrogen atoms were located in the difference electron density maps and refined with simple bond length constraints. Part of the molecule was disordered, with refined site occupancy factors of 0.698(4) for part A and 0.302(4) for part B. The structure was refined to *R* factor of 0.0367.

Complex **C1**: all non-hydrogen atoms were refined anisotropically. All hydrogen atoms, except the amino hydrogen (H3) on N3 and the hydroxyl hydrogen atoms of the methanol solvent molecules, were placed in idealized positions and refined in riding models with *U*_iso_ assigned 1.2 or 1.5 times *U*_eq_ of their parent atoms and the C–H bond distances were constrained from 0.95 Å to 0.99 Å. The other hydrogen atoms; H3, H1A, and H1B were located in the difference density maps and refined independently. The structure was refined to *R* factor of 0.0328.

Complex **C5**: all non-hydrogen atoms were refined anisotropically. All hydrogen atoms, except the amino hydrogen, were placed in idealized positions and refined in riding models with *U*_iso_ assigned 1.2 or 1.5 times *U*_eq_ of their parent atoms and the C–H bond distances were constrained from 0.95 Å to 0.99 Å. The hydrogen on N_2_ was located in the difference density maps and refined independently. The structure was refined to *R* factor of 0.0333.

### Synthesis of Precursors

Precursors **1** (*N* ^1^-(7-chloroquinolin-4-yl)-propane-1,3-diamine) and **2** (*N* ^1^-(2-aminoethyl)-*N* ^2^-(7-chloroquinolin-4-yl)­ethane-1,2-diamine) were synthesized following reported literature procedures.^72^

### General Method for Precursors **3** and **4**

Precursor **3** (*N* ^1^-(7-chloroquinolin-4-yl)-*N* ^3^-(4-methyl-2-nitrophenyl)-propane-1,3-diamine) was synthesized following a reported literature procedure.^71^ Briefly, 4-fluoro-3-nitrotoluene (1.5 equiv) was added to a stirring suspension of either **1** or **2** (1 equiv) in anhydrous DMF (8.00 mL). The resulting orange or yellow reaction mixture was stirred at room temperature, under Ar, for 24 h. Thereafter, 1 M NaOH (50.0 mL) was added to basify the mixture, which was stirred for approximately 10 min. The mixture was extracted with EtOAc (4 × 50.0 mL), the organic fractions combined, washed with brine (3 × 50.0 mL), and dried over anhydrous Na_2_SO_4_. Following filtration, the solvent was removed to afford the crude product as a bright orange residue, which was purified via column chromatography using DCM/MeOH (95:5) as the eluent.

Alternative method: 4-fluoro-3-nitrotoluene (1.2 equiv), K_2_CO_3_ (1.5 equiv), and Et_3_N (1.5 equiv) were added to a stirring solution of precursor **1** or **2** (1 equiv) in acetonitrile (10.0 mL). The mixture was heated to 75 °C and stirred overnight. Thereafter, the mixture was diluted with water (50.0 mL) and the aqueous solution extracted with EtOAc (3 × 100 mL). The organic fractions were combined, dried over anhydrous Na_2_SO_4_, filtered, and the solvent removed. The bright orange crude residue was then purified via column chromatography using DCM/MeOH (95:5) as the eluent.

### *N* ^1^-(7-Chloroquinolin-4-yl)-*N* ^2^-(2-((4-methyl-2-nitrophenyl)­amino)­ethyl)­ethane-1,2-diamine (**4**)

4-Fluoro-3-nitrotoluene (1.31 g, 8.41 mmol) and **2** (1.48 g, 5.61 mmol). Compound **4** was isolated as a red-orange powder. Yield: 33% (0.741 g, 1.85 mmol); *R*_f_ (DCM/MeOH, 9:1) = 0.40; LC–MS (*m*/*z*): 400.1 (M + H, calculated = 400.15, 100% purity; *t*_R_ = 0.53 min); ^1^H NMR (300 MHz, [D_4_]-MeOD, δ­(ppm)): 8.32 (d, 1H, H_a_, ^3^ *J*_H–H_ = 6.1 Hz), 8.09 (d, 1H, H_b_, ^3^ *J*_H–H_ = 9.0 Hz), 7.82 (br s, 1H, H_l_), 7.76 (d, 1H, H_c_, ^4^ *J*_H–H_ = 2.0 Hz), 7.42 (dd, 1H, H_d_, ^3,4^ *J*_H–H_ = 9.0 and 2.0 Hz), 7.25 (dd, 1H, H_k_, ^3,4^ *J*_H–H_ = 8.8 and 1.9 Hz), 6.86 (d, 1H, H_j_, ^3^ *J*_H–H_ = 8.8 Hz), 6.66 (d, 1H, H_e_, ^3^ *J*_H–H_ = 6.1 Hz), 3.58 (t, 2H, H_f_, ^3^ *J*_H–H_ = 6.0 Hz), 3.45 (t, 2H, H_i_, ^3^ *J*_H–H_ = 5.9 Hz), 3.11–2.96 (m, 4H, H_g,h_), 2.23 (s, 3H, H_m_); ^13^C­{^1^H} NMR (100 MHz, [D_6_]-DMSO, δ­(ppm)): 151.87, 150.10, 149.03, 143.63, 138.03, 133.31, 130.42, 127.44, 125.08, 124.10, 123.96, 117.41, 114.73, 98.68, 47.13, 46.75, 42.60, 42.06, 19.40; FT-IR (ATR): (*v*_max_/cm^–1^) = 3359 (N–H), 2917 (C–H), 2847 (C–H), 1609 (C=N), 1579 (C=C), 1524 (N–O), 1346 (N–O).

### General Method for Precursors **5** and **6**

Precursor **5** (*N* ^1^-(3-((7-chloroquinolin-4-yl)-amino)­propyl)-4-methylbenzene-1,2-diamine) was synthesized following a reported literature procedure.^71^ Briefly, NH_4_Cl (10 equiv) was added to a stirring solution of either **3** or **4** (1 equiv) in anhydrous MeOH (30.0 mL) and the mixture stirred for 30 min, at r.t., under Ar. Zn powder (20 equiv) was then added and the resulting gray mixture vigorously stirred for a further 24 h. Thereafter, 1 M NaOH (20.0 mL) was added to basify the mixture, which was filtered through Celite. The MeOH was removed under reduced pressure and the resulting aqueous solution extracted with DCM (3 × 50.0 mL). The organic fractions were collected, washed with brine (2 × 50.0 mL), followed by H_2_O (1 × 50.0 mL), and dried over anhydrous Na_2_SO_4_. Following filtration, the solvent of the filtrate was removed to afford the desired product.

### *N* ^1^-(2-((2-((7-Chloroquinolin-4-yl)­amino)­ethyl)­amino)­ethyl)-4-methylbenzene-1,2-diamine (**6**)

NH_4_Cl (0.261 g, 4.89 mmol), **4** (0.194 g, 0.486 mmol), and Zn powder (0.643 g, 9.83 mmol). Precursor **6** was isolated as a dark green powder. Yield: 77% (0.139 g; 0.376 mmol); LC–MS (*m*/*z*): 370.2 (M + H, calculated = 370.18, 100% purity; *t*_R_ = 0.29 min); ^1^H NMR (300 MHz, [D_4_]-MeOD, δ (ppm)): 8.30 (d, 1H, H_a_, ^3^ *J*_H–H_ = 5.6 Hz), 7.99 (d, 1H, H_b_, ^3^ *J*_H–H_ = 9.0 Hz), 7.74 (d, 1H, H_c_, ^4^ *J*_H–H_ = 1.9 Hz), 7.31 (dd, 1H, H_d_, ^3,4^ *J*_H–H_ = 9.0 and 2.0 Hz), 6.57–6.38 (m, 4H, H_e,j–l_), 3.42 (t, 2H, H_f_, ^3^ *J*_H–H_ = 6.3 Hz), 3.20 (t, 2H, H_i_, ^3^ *J*_H–H_ = 5.9 Hz), 2.92 (t, 2H, H_g_, ^3^ *J*_H–H_ = 6.3 Hz), 2.87 (t, 2H, H_h_, ^3^ *J*_H–H_ = 5.9 Hz), 2.13 (s, 3H, H_m_); ^13^C­{^1^H} NMR (100 MHz, [D_4_]-MeOD, δ (ppm)): 152.60, 152.22, 149.34, 136.32, 136.27, 135.50, 129.29, 127.39, 126.03, 124.30, 121.25, 118.67, 118.23, 113.50, 99.66, 49.20, 48.13, 44.53, 43.17, 20.81; FT-IR (ATR): (*v*_max_/cm^–1^) = 3301 (N–H), 2915 (C–H), 2847 (C–H), 1609 (C=N), 1579 (C=C).

### Synthesis of Hybrid Complexes

#### General Method for Complexes **C1**–**C4**

Complex **C1** (7-chloro-*N*-(3-(5-(methyl)-2-ferrocenyl-1*H*-benzo­[*d*]­imidazole-1-yl)­propyl)­quinolin-4-amine) was synthesized following a reported literature procedure.^71^ Briefly, TFA (0.1 equiv) was added to a stirring solution of either ferrocenecarboxaldehyde or 2-(*N*,*N*-dimethylaminomethyl)­ferrocenecarboxaldehyde (1.1 equiv) in EtOH (20.0 mL) and the solution stirred for 5 min. Thereafter, either precursor **5** or **6** (1 equiv) and anhydrous MgSO_4_ (5 equiv) were added, and the dark red reaction mixture refluxed, at 75 °C, for 48 h. The mixture was then filtered by gravity, to remove the MgSO_4_, and the solvent of the filtrate removed to give a dark red residue. To the crude reside, 1 M NaOH (50.0 mL) was added and the aqueous solution extracted with DCM (3 × 50.0 mL). The organic fractions were combined, washed with brine (2 × 50.0 mL), followed by H_2_O (1 × 50.0 mL), and dried over anhydrous Na_2_SO_4_. The solvent of the filtrate was removed to afford the crude product, as a dark red oil, which was purified via column chromatography.

#### *N* ^1^-(7-Chloroquinolin-4-yl)-*N* ^2^-(2-(5-methyl-2-ferrocenyl-1*H*-benzo­[*d*]­imidazole-1-yl)­ethyl)­ethane-1,2-diamine (**C2**)

TFA (2.33 μL, 0.0304 mmol), ferrocenecarboxaldehyde (0.0651 g, 0.304 mmol), compound **6** (0.112 g, 0.304 mmol), and anhydrous MgSO_4_ (0.183 g, 1.52 mmol). The crude product was purified via column chromatography, using DCM/MeOH as the eluent, and **C2** was isolated as a brown-red powder. Yield: 35% (59.6 mg; 0.106 mmol); *R*_f_ (DCM/MeOH, 95:5) = 0.07 (0.18 after 2 MD); LC–MS (*m*/*z*): 282.6 ([M + 2H]^2+^, calculated = 282.6), 564.1 ([M + H]^+^, calculated = 564.2, 100% purity; *t*_R_ = 0.63 min); HPLC: purity = 99% (*t*_R_ = 2.98 min); ^1^H NMR (300 MHz, [D_4_]-MeOD, δ (ppm)): 8.32 (d, 1H, H_a_, ^3^ *J*_H–H_ = 5.7 Hz), 7.91 (d, 1H, H_b_, ^3^ *J*_H–H_ = 9.0 Hz), 7.79 (d, 1H, H_c_, ^4^ *J*_H–H_ = 1.4 Hz), 7.44–7.31 (m, 3H, H_d‑f_), 7.01 (d, 1H, H_g_, ^3^ *J*_H–H_ = 8.4 Hz), 6.51 (d, 1H, H_h_, ^3^ *J*_H–H_ = 5.7 Hz), 4.96 (br s, 2H, H_j_), 4.61 (t, 2H, H_o_, ^3^ *J*_H–H_ = 6.9 Hz), 4.43 (br s, 2H, H_k_), 4.14 (s, 5H, H_q_), 3.45 (t, 2H, H_l_, ^3^ *J*_H–H_ = 6.0 Hz), 3.15 (t, 2H, H_n_, ^3^ *J*_H–H_ = 6.9 Hz), 2.99 (t, 2H, H_m_, ^3^ *J*_H–H_ = 6.1 Hz), 2.42 (s, 3H, H_p_); ^13^C­{^1^H} NMR (100 MHz, [D_4_]-MeOD, δ (ppm)): 154.73, 152.77, 152.28, 149.42, 143.74, 136.47, 135.33, 133.46, 127.48, 126.20, 124.99, 124.20, 118.74, 118.64, 110.47, 99.82, 74.25, 71.40, 70.77, 70.29, 49.60, 48.63, 45.51, 43.33, 21.60; FT-IR (ATR): (*v*_max_/cm^–1^) = 3251 (N–H), 1611 (C=N), 1578 (C=C); HR-MS (ESI (+), *m*/*z*): found = 564.1555 (98%, [M + H]^+^), calculated = 564.1612.

#### 7-Chloro-*N*-(3-(5-methyl-2-(2-*N*,*N*-dimethylaminomethyl)­ferrocenyl-1H-benzo­[*d*]-imidazole-1-yl)­propyl) Quinoline-4-amine (**C3**)

TFA (5.00 μL, 0.0654 mmol), 2-(*N*,*N*-dimethylaminomethyl)­ferrocenecarboxaldehyde (0.266 g, 0.980 mmol), precursor **5** (0.223 g, 0.654 mmol), and MgSO_4_ (0.394 g, 3.27 mmol). The reaction mixture was refluxed for 72 h and the crude product purified via column chromatography using a mixture of petroleum ether, EtOAc, and Et_3_N as the eluent. Complex **C3** was isolated as a dark yellow powder. Yield: 29% (0.114 g; 0.192 mmol); HPLC: purity = 98% (*t*_R_ = 2.03 min); ^1^H NMR (600 MHz, [D_4_]-MeOD, δ (ppm)): 8.33 (d, 1H, H_a_, ^3^ *J*_H–H_ = 5.5 Hz), 7.96 (d, 1H, H_b_, ^3^ *J*_H–H_ = 8.9 Hz), 7.81 (d, 1H, H_f_, ^4^ *J*_H–H_ = 1.6 Hz), 7.49 (br s, 1H, H_c_), 7.42 (dd, 1H, H_e_, ^3,4^ *J*_H–H_ = 8.9 and 1.5 Hz), 7.34 (d, 1H, H_d_, ^3^ *J*_H–H_ = 8.2 Hz), 7.05 (d, 1H, H_g_, ^3^ *J*_H–H_ = 8.1 Hz), 6.46 (d, 1H, H_h_, ^3^ *J*_H–H_ = 5.5 Hz), 4.43 (br s, 1H, H_i_), 4.45–4.39 (m, 1H, H_j_), 4.42 (br s, 1H, H_p_), 4.35–4.27 (m, 1H, H_j′_), 4.18 (s, 5H, H_l_), 4.15 (d, 1H, H_r_, ^2^ *J*_H–H_ = 13.3 Hz), 3.96 (br s, 1H, H_k_), 3.52 (t, 2H, H_m_, ^3^ *J*_H–H_ = 5.9 Hz), 3.42 (d, 1H, H_r′_, ^2^ *J*_H–H_ = 13.3 Hz), 2.45 (s, 3H, H_n_), 2.29–2.19 (m, 1H, H_o_), 2.19–2.09 (m, 1H, H_o′_), 1.98 (s, 6H, H_q_); ^13^C­{^1^H} NMR (100 MHz, [D_4_]-MeOD, δ (ppm)): 153.18, 152.50, 152.34, 149.82, 143.89, 136.46, 134.58, 133.37, 127.72, 126.13, 125.17, 124.38, 119.30, 118.89, 110.60, 99.90, 76.45, 73.32, 71.80, 69.62, 69.43, 57.37, 44.37, 43.55, 40.84, 29.22, 21.63; FT-IR (ATR): (*v*_max_/cm^–1^) = 3240 (N–H), 1614 (C=N); HR-MS (ESI (+), *m*/*z*): found = 296.6024 (80%, [M + 2H]^2+^), calculated = 296.6002; found = 592.1934 (97%, [M + H]^+^), calculated = 592.1925.

#### *N* ^1^-(7-Chloroquinolin-4-yl)-*N* ^2^-(2-(5-methyl-2-(2-*N*,*N*-dimethylaminomethyl)­ferrocenyl-1*H*-benzo­[*d*]­imidazole-1-yl)­ethyl)­ethane-1,2-diamine (**C4**)

TFA (5.00 μL, 0.0654 mmol), 2-(*N*,*N*-dimethylaminomethyl)­ferrocenecarboxaldehyde (0.577 g, 2.13 mmol), precursor 6 (0.477 g, 1.29 mmol), and MgSO_4_ (1.05 g, 8.73 mmol). The reaction mixture was refluxed for 92.5 h and the crude product purified via column chromatography using a mixture of Et_3_N, DCM and MeOH as the eluent. Complex C4 was isolated as a dark brown powder. Yield: 20% (0.158 g; 0.255 mmol); LC–MS (*m*/*z*): 311.1 ([M+2H]^2+^, calculated = 311.1), 621.2 ([M + H]^+^, calculated = 621.2, 98% purity; *t*_R_ = 0.64 min); ^1^H NMR (600 MHz, [D_4_]-MeOD, δ (ppm)): 8.32 (d, 1H, H_a_, ^3^ *J*_H–H_ = 5.6 Hz), 7.94 (d, 1H, H_b_, ^3^ *J*_H–H_ = 9.0 Hz), 7.76 (d, 1H, H_c_, ^4^ *J*_H–H_ = 1.4 Hz), 7.48 (s, 1H, H_f_), 7.43–7.33 (m, 2H, H_d,e_), 7.05 (d, 1H, H_g_, ^3^ *J*_H–H_ = 8.2 Hz), 6.46 (d, 1H, H_h_, ^3^ *J*_H–H_ = 5.6 Hz), 4.80 (br s, 1H, H_k_), 4.54 (br s, 1H, H_j_), 4.36 (br s, 1H, H_i_), 4.50–4.40 (m, 1H, H_o_), 4.34–4.27 (m, 1H, H_o_), 4.23 (s, 5H, H_q_), 4.17 (d, 1H, H_r_, ^2^ *J*_H–H_ = 13.2 Hz), 3.52 (d, 1H, H_r′_, ^2^ *J*_H–H_ = 13.3 Hz), 3.40 (t, 2H, H_l_, ^3^ *J*_H–H_ = 5.8 Hz), 3.03 (t, 2H, H_n_, ^3^ *J*_H–H_ = 6.8 Hz), 2.97–2.88 (m, 2H, H_m_), 2.45 (s, 3H, H_p_), 2.07 (s, 6H, H_s_); ^13^C­{^1^H} NMR (100 MHz, [D_4_]-MeOD, δ (ppm)): 153.38, 152.60, 152.52, 149.62, 143.62, 136.33, 134.77, 133.50, 127.62, 126.09, 125.37, 124.27, 119.19, 118.74, 110.78, 99.70, 76.46, 73.59, 71.92, 71.86, 70.37, 69.92, 57.44, 53.50, 45.36, 43.88, 43.24, 21.64; FT-IR (ATR): (*v*_max_/cm^–1^) = 3255 (N–H), 1611 (C=N); HR-MS (ESI (+), *m*/*z*): found = 621.2208 (100%, [M + H]^+^), calculated = 621.2190.

#### *N* ^1^-Ferrocenyl-*N* ^2^-(7-Chloroquinolin-4-yl)-N1-(2-(5-methyl-2-ferrocenyl-1*H*-benzo­[*d*]­imidazole-1-yl)­ethyl)­ethane-1,2-diamine (**C5**)

Precursor **6** (0.222 g, 0.601 mmol), ferrocenecarboxaldehyde (0.193 g, 0.901 mmol), TFA (4.60 μL, 0.0601 mmol) and MgSO_4_ (0.366 g, 3.04 mmol). The dark red reaction mixture was refluxed for 72 h. The crude product was purified using a mixture of petroleum ether, EtOAc, and Et_3_N as the eluent. Complex **C5** was isolated as a dark orange powder. Yield: 34% (0.116 g; 0.206 mmol); *R*_f_ (EtOAc/pet ether/Et_3_N, 7:2:1) = 0.43; HPLC: purity = 97% (*t*_R_ = 2.96 min); ^1^H NMR (300 MHz, [D_6_]-DMSO, δ (ppm)): 8.37 (d, 1H, H_a_, ^3^ *J*_H–H_ = 5.3 Hz), 8.12 (d, 1H, H_b_, ^3^ *J*_H–H_ = 9.1 Hz), 7.79 (d, 1H, H_c_, ^4^ *J*_H–H_ = 2.1 Hz), 7.45 (dd, 1H, H_d_, ^3,4^ *J*_H–H_ = 8.9 and 2.1 Hz), 7.39–7.31 (m, 2H, H_e,f_), 7.05–6.95 (m, 2H, H_g,_ NH), 6.32 (d, 1H, H_h_, ^3^ *J*_H–H_ = 5.5 Hz), 4.79 (br s, 2H, H_i_), 4.47 (t, 2H, H_o_, ^3^ *J*_H–H_ = 5.8 Hz), 4.30 (br s, 2H, H_i_), 4.19 (br s, 2H, H_j_), 4.12 (br s, 2H, H_j_), 4.08 (s, 5H, H_k_), 4.06 (s, 5H, H_k_), 3.56 (s, 2H, H_q_), 3.25–3.14 (m, 2H, H_l_), 2.84 (t, 2H, H_m_, ^3^ *J*_H–H_ = 6.4 Hz), 2.71 (t, 2H, H_n_, ^3^ *J*_H–H_ = 5.8 Hz), 2.38 (s, 3H, H_p_); ^13^C­{^1^H} NMR (100 MHz, [D_6_]-DMSO, δ (ppm)): 152.30, 151.94, 149.87, 149.07, 143.07, 134.13, 133.41, 130.59, 127.58, 124.15, 123.84, 122.93, 118.09, 117.42, 109.63, 98.83, 82.90, 74.27, 69.95, 69.62, 69.33, 69.02, 68.40, 67.86, 53.49, 52.22, 51.77, 43.61, 40.69, 21.20; FT-IR (ATR): (*v*_max_/cm^–1^) = 3350 (N–H), 1609 (C=N); HR-MS (ESI (+), *m*/*z*): found = 381.5911 (100%, [M + 2H]^2+^), calculated = 381.5908; found = 564.1613 (100%, [M – Fc­(CH_2_) + 2H]^+^), calculated = 564.1612.

### In Vitro Blood Stage Antiplasmodial Activity

Test samples were screened in technical duplicate or triplicate, for three or four biological replicates, for their in vitro blood stage antiplasmodial activity against both the drug-sensitive (NF54) and multidrug-resistant (K1) strains of . Continuous in vitro cultures of asexual erythrocyte stages of were maintained using a modified method of Trager and Jensen.^110^ A quantitative assessment of the in vitro antiplasmodial activity was determined via the parasite lactate dehydrogenase (*p*LDH) assay, using a modified method described by Makler and Hinrichs.^79^

The test samples were prepared to a 20 mg/mL stock solution in 100% DMSO. Stock solutions were stored at −20 °C. Further dilutions were prepared in complete medium on the day of the experiment. Samples were tested as a suspension if not completely dissolved, as was the case for **C5**. Chloroquine diphosphate (CQDP) was used as the reference drug in all experiments. Test samples were tested at a starting concentration of either 100 μg/mL, 50 μg/mL, 10 μg/mL, or 1 μg/mL, which was then serially diluted 2-fold, in complete medium, to give 10 concentrations. The same dilution technique was used for all samples. A full dose–response was performed for all compounds to determine the concentration inhibiting 50% of parasite growth (IC_50_ value), following 48 h of incubation. The IC_50_ values were obtained from full dose–response curves, using a nonlinear dose–response curve fitting analysis, via GraphPad Prism v.5 software.

### Selectivity Studies

The samples were tested over 48 h against the Chinese hamster ovarian (CHO) cell line to determine general cytotoxicity. Continuous cultures of CHO cells were maintained using the method described by Mosmann^111^ with minor modifications. Quantitative assessment of toxic activity in vitro was determined via the MTT assay using the method described. Cell viability was determined colorimetrically using the breakdown of a dye by enzymes of the electron transport chain, taking place in the mitochondria of metabolically active living cells, as a marker for survival.

Compounds were tested in triplicate on a single occasion against these cells using the MTT assay. The test samples were prepared to a 10 mM stock solution, in 100% DMSO, and stored at −20 °C until use. Samples were tested as a suspension if not completely dissolved. Further dilutions to the desired starting concentration were freshly prepared in growth media at the start of the experiment.

Cells were plated to a density of 105 cells/well in 96-well plates and allowed to attach for 24 h. Thereafter, compounds were added at various concentrations from 50 μmol/L down to 16 nmol/L and the cells incubated for a further 48 h. Emetine was used as the control compound since it shows nonspecific cytotoxicity to mammalian cells. After 44 h MTT was added, and the plates were read 4 h later at 540 nm on a spectrophotometer following the addition of 100 μL of DMSO and gentle plate-shaking to solubilize and visualize the dye. Survival was plotted against concentration, and the IC_50_ values were obtained using a nonlinear dose–response curve fitting analysis via the Dotmatics software platform.

### In Vitro Gametocytocidal Antiplasmodial Activity

Compounds are assayed on the luciferase reporter line (NF54-*Pfs*16-GFP-Luc) assay, which allows for stage-specific determination of gametocytocidal action on immature (>90% stage II/III) and mature (>95% stage V) stage gametocytes.

The luciferase reporter assay was established to enable accurate, reliable and quantifiable investigations of the stage-specific action of gametocytocidal compounds for both immature and mature gametocytes using the marker cell line; NF54-*Pf*s16-GFP-Luc.^112,113^ Drug assays were set up on day 5 and 13 (representing >90% of either immature stage II/III or >95% mature stage V gametocytes, respectively). In each instance, assays were set up using a 2–3% gametocytaemia, 1.5% hematocrit culture and 48 h drug pressure under hypoxic conditions (90% N_2_, 5% O_2_, and 5% CO_2_) at 37 °C, without shaking. Compounds are diluted 3-fold in at least a 9-point serial dilution series starting at 20 μM, with a nonlethal DMSO concentration of <0.1% maintained. Luciferase activity was determined in 30 μL parasite lysates by adding 30 μL luciferin substrate (Promega Luciferase Assay System) at room temperature and detection of resultant bioluminescence at an integration constant of 10 s with the GloMax-Multi + Detection System with Instinct Software. Methylene blue (5 μM) and the clinical antimalarial candidate MMV390048 (5 μM) were used as controls. IC_50_ values were determined in GraphPad Prism using a nonlinear regression fit with variable slope (4-parameter) on the dose–response data. All IC_50_ values are reported after three independent biological repeats (*n* = 3), each performed in technical triplicates, with data reported as average ±SE.

Assay quality parameters are evaluated for each assay, including Z′-factors (data quality compared to reproducibility using signal, noise, and background parameters; accepted metric >0.6), regression (accepted *r*_2_ > 0.9); >80% viable parasites (top plateau); <20% killed parasites (bottom plateau), hill slope ∼1. Internal controls should perform at >90% inhibition for methylene blue and >70% inhibition for MMV390048.

### β-Hematin Formation Inhibition Assay

The β-hematin formation inhibition assay was modified from the method reported by Sandlin et al.^91,97^ Stock solutions of the respective test compounds were prepared to 10 mM, in 100% DMSO, with the exception of CQ (used in the salt form, CQDP), which was prepared in Milli-Q water. A 100 μL aliquot of a water/NP-40 (305.5 μM)/DMSO solution, in a v/v ratio of 7/2/1, was added to all wells in columns 1–11. Milli-Q water (140 μL), NP-40 (305.5 μM, 40 μL), and a 20 μL aliquot of the test compound were added to the wells in column 12. NP-40 is a detergent used to mediate the formation of β-hematin. The solution in column 12 was then serially diluted 2-fold, through to column 2, to give a total of 11 concentrations. Column 1 contained 0 μM of the test compound and thus served as a blank. A 25 mM stock solution of hematin was prepared by dissolving 16.3 mg of hemin in 1.0 mL of DMSO and sonicated for 1 min. A 178.8 μL aliquot of this hematin suspension was added to 20.0 mL of a 2 M acetate buffer (pH = 4.8) and 100 μL of this solution added to each well. The plates were then covered and incubated for 5 h, at 37 °C. The assay was analyzed using the pyridine-ferrochrome method developed by Ncokazi and Egan.^114^ A solution containing water/acetone/2 M HEPES buffer (pH 7.4)/pyridine, in a v/v ratio of 2/2/1/5, was prepared and 32 μL was added to all wells, followed by 60 μL of acetone. The absorbance values were then measured at 405 nm, the results plotted using GraphPad Prism (v5), and the IC_50_ values obtained using a sigmoidal dose–response curve fitting analysis.

### Cellular Heme Fractionation Studies

Compound **C3** was tested in the heme fractionation assay, using a previously reported method,^100^ to determine intracellular levels of free heme and hemozoin. In a 24-well plate, ring-stage *Pf*NF54 parasites, at 5% parasitemia and 2% hematocrit, were synchronized and treated with various concentrations of the test compound (multiples of the IC_50_ value). The IC_50_ was previously determined using the *p*LDH assay. Following incubation for 28–32 h, to ensure progression to the trophozoite stage, parasitized red blood cells were saponin lysed to obtain isolated trophozoites. To determine the amount of heme and hemozoin, the trophozoites were then exposed to a series of cellular fractionation steps to successively liberate each heme species. The UV–visible spectra of these fractions were recorded between 400 and 415 nm on a multiwell plate reader. The percentage of each heme species was determined from the absorbance value of the Soret band of the pyridyl heme complex. To determine the absolute levels of each species, an aliquot of the isolated trophozoites was spiked with Trucount beads and stained with SYBR Green. These were run on a flow cytometer to determine absolute counts, which could in turn be used to calculate the absolute levels of each species per cell. The total heme in each of these samples was quantified using a standard curve. GraphPad Prism (v5) was used to analyze the final data set and perform the significance tests.

### Reactive Oxygen Species Generation

Stock solutions of DCFH_2_-DA were prepared to 1 mM in 100% DMSO and stored at −20 °C in the dark until use. The deacetylation of DCFH_2_-DA involved adding 2 μL of a 0.5 M NaOH solution to 200 μL of the 1 mM DCFH_2_-DA stock solution. The solution was mixed for 30 min, in the dark, at room temperature and then quenched with 800 μL of PBS buffer to obtain a 200 μM H_2_DCF 1:4 DMSO/PBS solution mixture.

The complex solutions (**C1**–**C5**, **Fc** and **FQ**) were prepared to 200 μM in a 1:4 DMSO/PBS solution mixture. The fluorometer was blanked using a 1:4 DMSO/PBS solution mixture. To identify hydroxyl radical production, 2 μL of a 0.5% H_2_O_2_ solution was added to 900 μL of the 200 μM complex solution in a cuvette at 25 °C. 100 μL of the prepared H_2_DCF solution (200 μM) was quickly added, and the readings recorded at 530 nm (emission wavelength), at 2 min intervals, over 30 min.

## Supplementary Material



## Notes

^1^H and ^13^C­{^1^H} NMR spectra, HPLC and LC–MS traces, cyclic voltametric and crystallographic data, and fluorescence emission spectra for all compounds (PDF)

## Abbreviations

WHO: World Health Organization
ACT: artemisinin-based combination therapy
MMV: medicines for malaria ventures
Hz: hemozoin
Hb: hemoglobin
FQ: ferroquine
CQ: chloroquine
Cp: cyclopentadienyl
DV: digestive vacuole
MDR: multidrug-resistant
ABS: asexual blood stage
S_N_Ar: nucleophilic aromatic substitution
LC-MS: liquid chromatography–mass spectrometry
TFA: trifluoroacetic acid
HR-MS: high resolution-mass spectrometry
HPLC: high-performance liquid chromatography
3D: 3-dimensional
*Pf*CRT: chloroquine resistance transporter
*p*LDH: lactate dehydrogenase
CQS: chloroquine-sensitive
CQR: chloroquine-resistant
CQDP: chloroquine diphosphate
RI: resistance index
SI: selectivity index
ROS: reactive oxygen species
GSH: glutathione
CHO: Chinese hamster ovary
DCF: 2′,7′-dichlorofluorescein
TLC: thin layer chromatography
TMS: trimethylsilane
IR: infrared
