# Supplementary material for: Ferrocenyl Quinoline-Benzimidazole Hybrids: A Multistage Strategy to Combat Drug-Resistant Malaria
Source: Inorg Chem. 2025 Jul 31;64(31):16152–67. doi: 10.1021/acs.inorgchem.5c02689 (PMC12344774; doi:10.1021/acs.inorgchem.5c02689)
Supplement: Supplementary file 1 [file ic5c02689_si_001.pdf]

Supporting Information

Ferrocenyl Quinoline-Benzimidazole Hybrids: A Multistage Strategy to Combat Drug-Resistant Malaria

Taryn M. Golding[a], Larnelle F. Garnie[a], Tayla Rabie[b], Janette Reader[b], Lyn-Marié Birkholtz[b,c], Kathryn J. Wicht[a,d,e] and Gregory S. Smith[a,*]

[a] Department of Chemistry, University of Cape Town, Rondebosch, Cape Town, 7701, South Africa.

[b] Department of Biochemistry, Genetics and Microbiology, Institute for Sustainable Malaria Control, University of Pretoria, Hatfield, 0028, South Africa.

[c] Department of Biochemistry, Stellenbosch University, Matieland, Stellenbosch, 7602, South Africa.

[d] Holistic Drug Discovery and Development (H3D) Centre, University of Cape Town, Rondebosch, Cape Town, 7701, South Africa.

[e] South African Medical Research Council Drug Discovery and Development Research Unit, Institute of Infectious Disease and Molecular Medicine, University of Cape Town, Rondebosch, Cape Town, 7701, South Africa.

*Gregory.Smith@uct.ac.za

Figure S1. ^{1}H NMR spectra of precursors **1**, **3**, and **5** S3

Figure S2. ^{1}H NMR spectra of precursors **2**, **4**, and **6** S3

Figure S3. ^{13}C{^{1}H} NMR spectrum of precursor **4** S4

Figure S4. ^{13}C{^{1}H} NMR spectrum of precursor **6** S4

Figure S5. LC-MS trace of precursor **4** S5

Figure S6. Mass spectrum of precursor **4** S6

Figure S7. LC-MS trace of precursor **6** S7

Figure S8. Mass spectrum of precursor **6** S8

Figure S9. ^{1}H NMR spectrum of complex **C1** S9

Figure S10. ^{1}H NMR spectrum of complex **C2** S9

Figure S11. ^{1}H NMR spectrum of complex **C3** S10

Figure S12. ^{1}H NMR spectrum of complex **C4** S10

Figure S13. ^{1}H NMR spectrum of complex **C5** S11

Figure S14. ^{13}C{^{1}H} NMR spectrum of complex **C2** S12

Figure S15. ^{13}C{^{1}H} NMR spectrum of complex **C3** S12

Figure S16. ^{13}C{^{1}H} NMR spectrum of complex **C4** S13

Figure S17. $^{13}C\{^1H\}$ NMR spectrum of complex **C5** S13
Figure S18. HPLC trace of complex **C1** S14
Figure S19. HPLC trace of complex **C2** S14
Figure S20. LC trace of complex **C2** S15
Figure S21. Mass spectrum of complex **C2** S15
Figure S22. HPLC trace of complex **C3** S16
Figure S23. LC trace of complex **C4** S17
Figure S24. Mass spectrum of complex **C4** S18
Figure S25. HPLC trace of complex **C5** S18
Figure S26. HR Mass spectra of complex **C1** S19
Figure S27. HR Mass spectrum of complex **C2** S20
Figure S28. HR Mass spectrum of complex **C3** S21
Figure S29. HR Mass spectrum of complex **C4** S22
Figure S30. HR Mass spectrum of complex **C5** S22
Figure S31. Molecular structure of precursor **4** S23
Table S1. Crystallographic data and refinement parameters for precursor **4** S23
Table S2. Crystallographic data and refinement parameters for complex **C1** S24
Table S3. Selected bond lengths (Å) and angles (°) for complex **C1** S24
Table S4. Crystallographic data and refinement parameters for complex **C5** S24
Table S5. Selected bond lengths (Å) and angles (°) for complex **C5** S25
Table S6. Cyclic voltametric parameters of complexes **C1** – **C5** in CH_2Cl_2 .... S25
Figure S32. Cyclic voltammograms of complexes **C1** – **C5** in CH_2Cl_2 .... S25
Figure S33. Fluorescence emission spectra of H_2O_2, ferrocene and FQ S26
Figure S34. Fluorescence emission spectra of complexes **C1**, **C3**, and **C5** S27
Figure S35. Stability of **C1**, in DMSO, over 48 hours S28
Figure S36. Stability of **C5**, in DMSO, over 48 hours S28

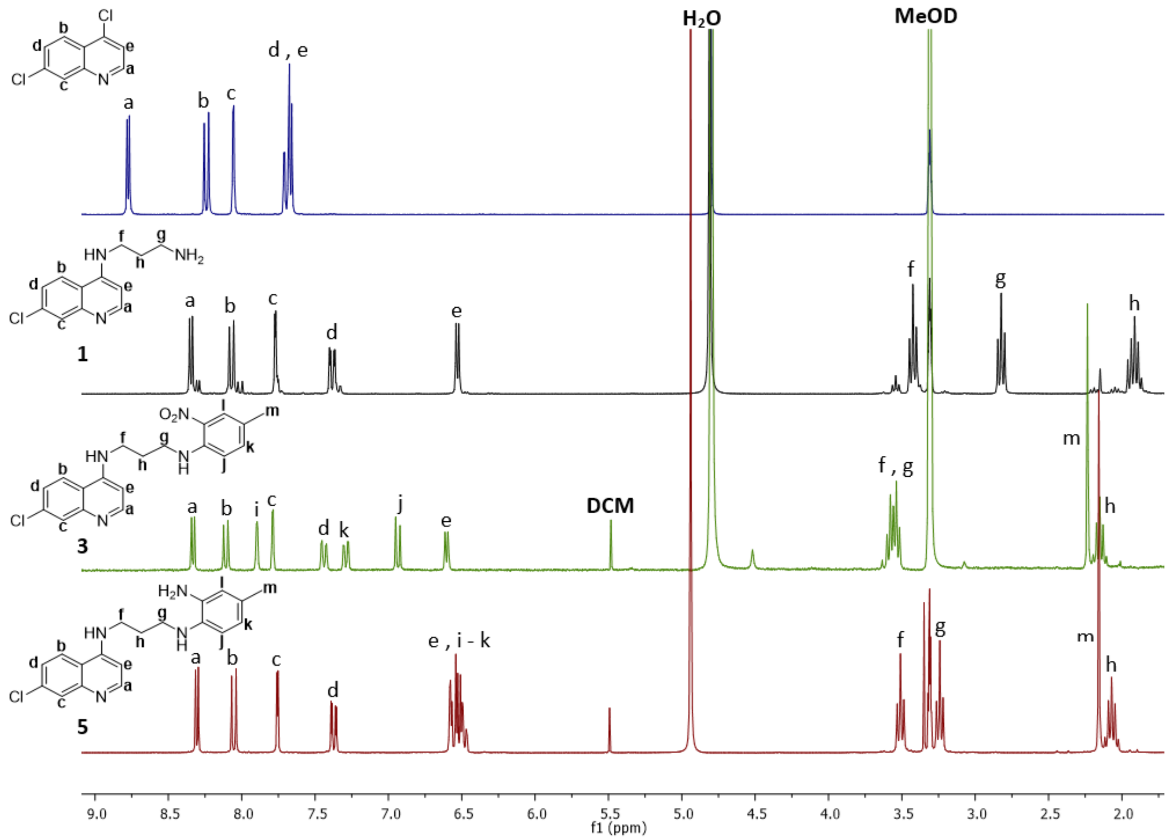

Figure S1. Stacked ^{1}H NMR spectra of 4,7-dichloroquinoline and the aminoquinoline precursors **1**, **3**, and **5** in MeOD.

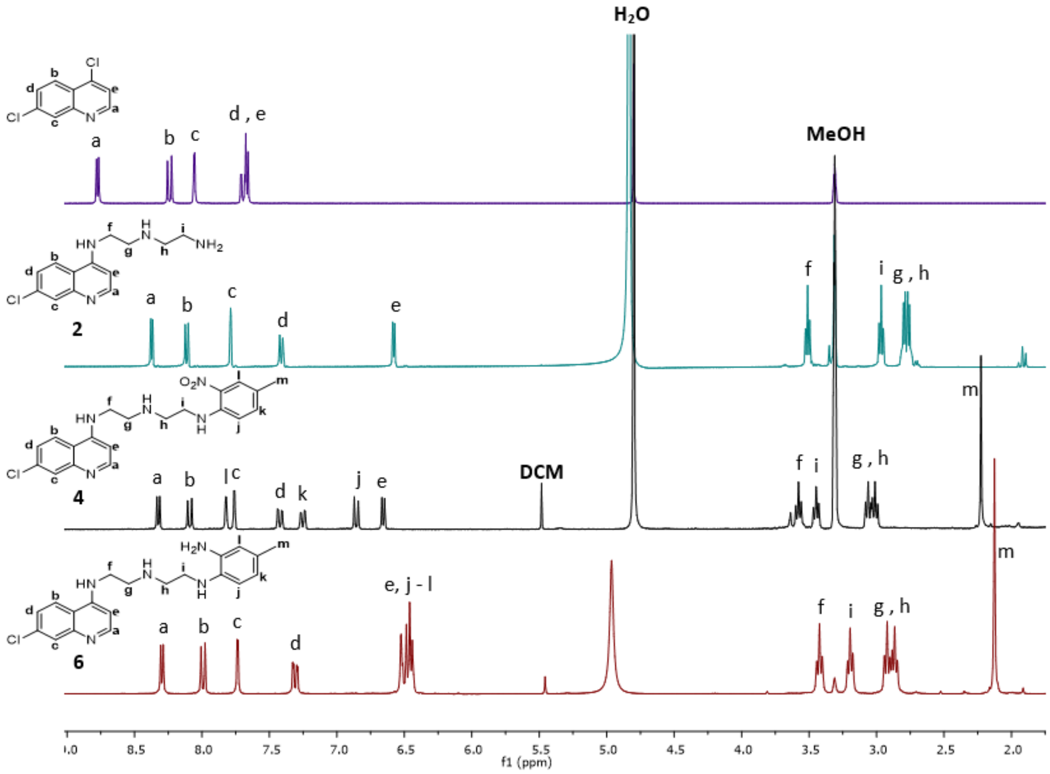

Figure S2. Stacked ^{1}H NMR spectra of 4,7-dichloroquinoline and the aminoquinoline precursors **2**, **4**, and **6** in MeOD.

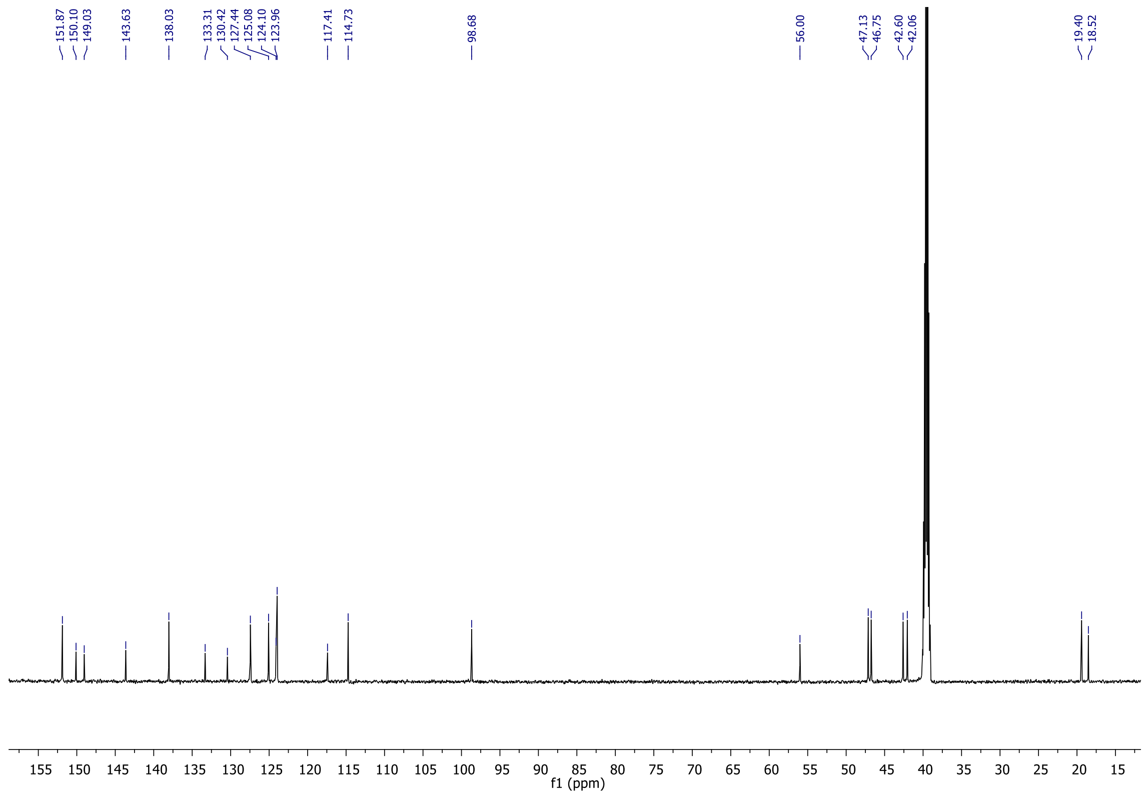

Figure S3. $^{13}C\{^1H\}$ NMR spectrum of precursor **4**, in DMSO.

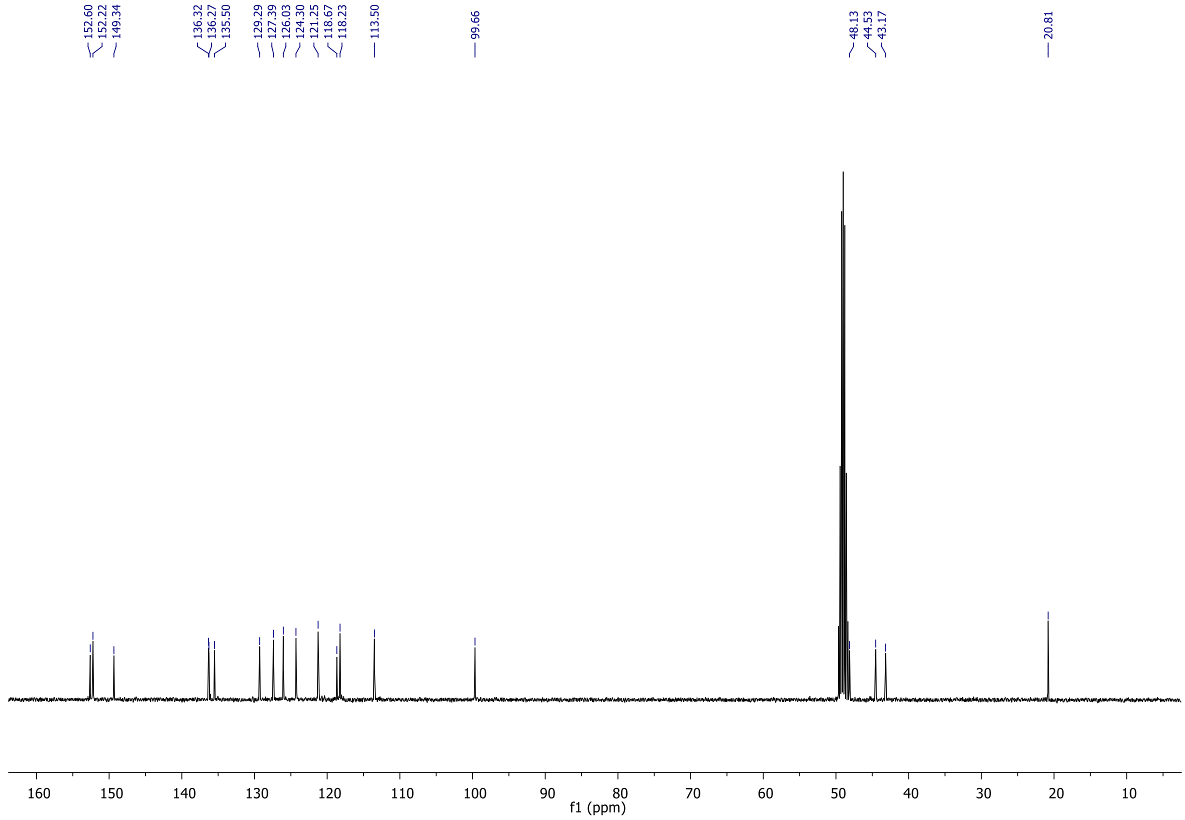

Figure S4. $^{13}C\{^1H\}$ NMR spectrum of precursor **6**, in MeOD.

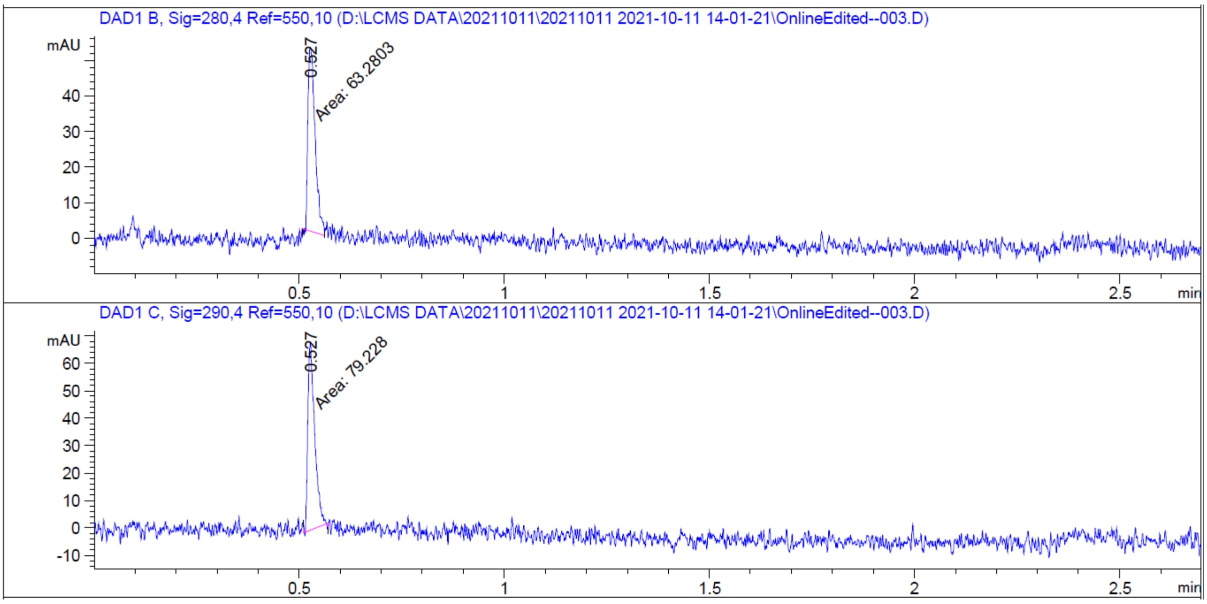

Signal 2: DAD1 B, Sig=280,4 Ref=550,10

Peak #	RetTime [min]	Type	Width [min]	Area [mAU*s]	Height [mAU]	Area %
1	0.527	MM	0.0204	63.28032	51.78416	100.0000
Totals :				63.28032	51.78416	

Signal 3: DAD1 C, Sig=290,4 Ref=550,10

Peak #	RetTime [min]	Type	Width [min]	Area [mAU*s]	Height [mAU]	Area %
1	0.527	MM	0.0192	79.22803	68.93510	100.0000
Totals :				79.22803	68.93510	

Figure S5. LC-MS trace and peak area for compound **4**.

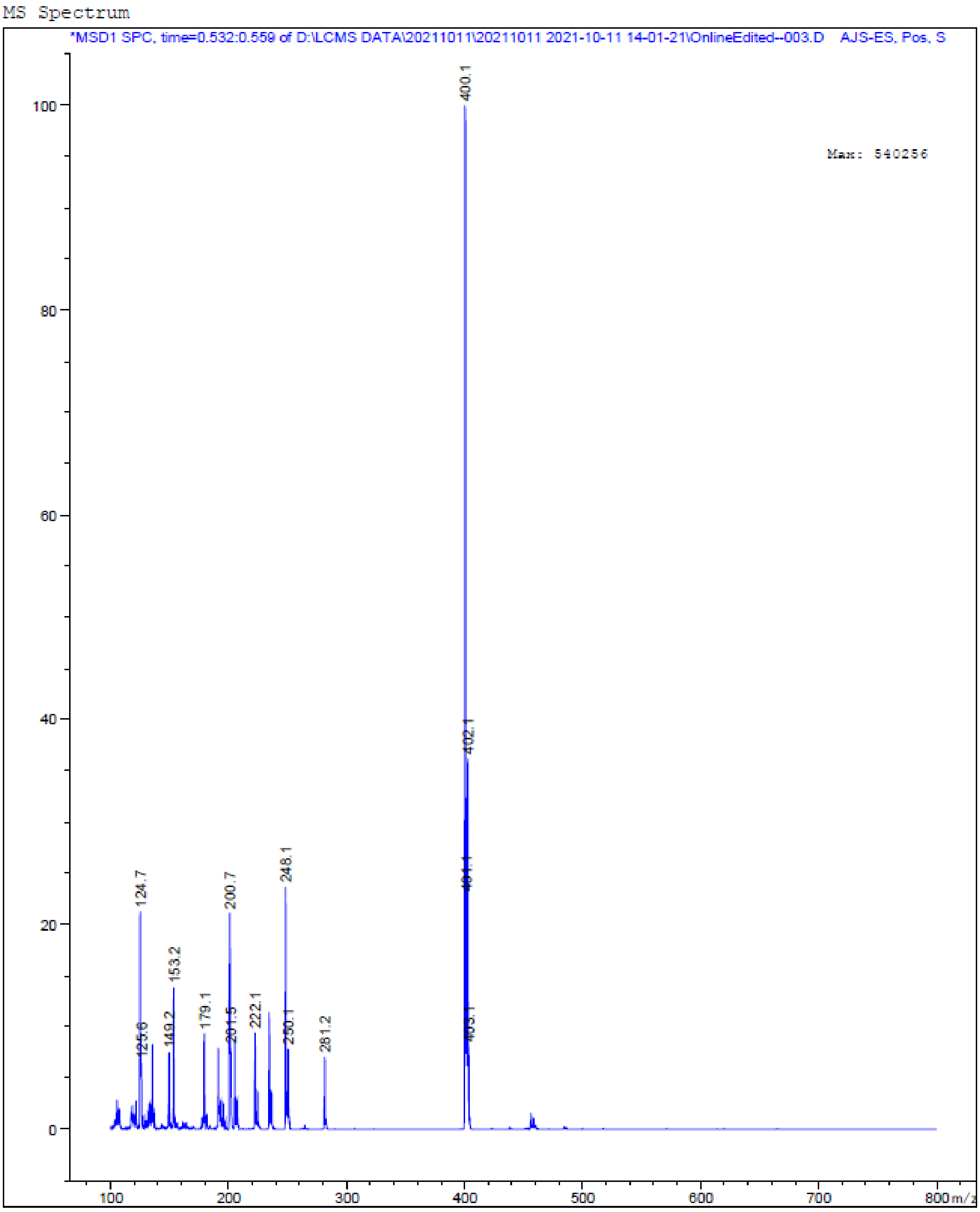

Figure S6. Mass spectrum of compound **4**.

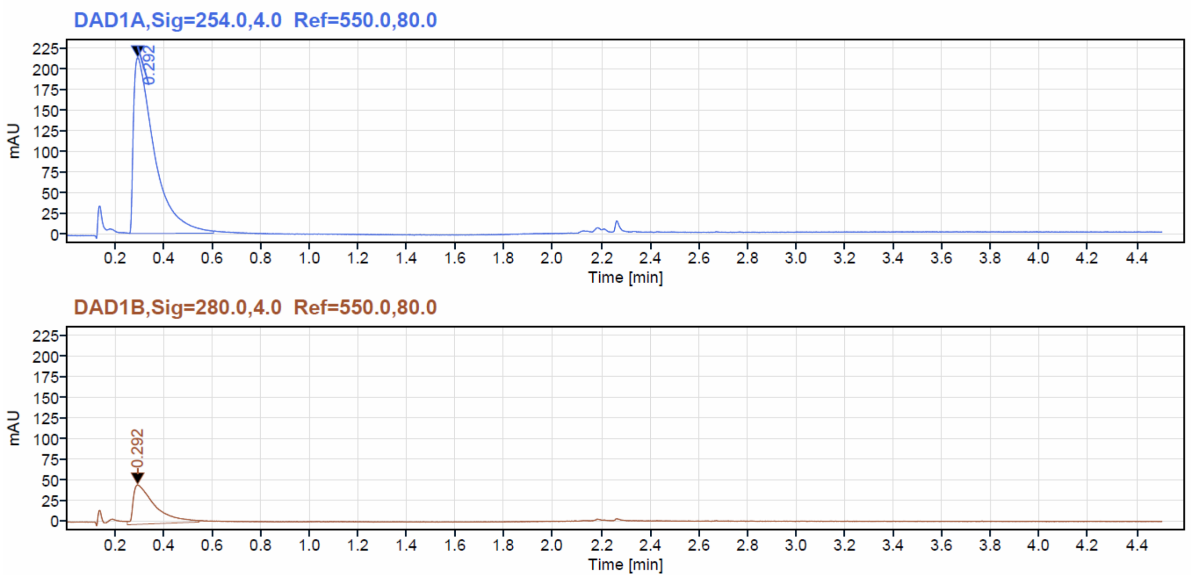

Signal: DAD1A,Sig=254.0,4.0 Ref=550.0,80.0

RT [min]	Type	Width [min]	Area	Height	Area%
0.292	BV	0.35	1264.27	214.14	100.00
		**Sum**	**1264.27**		

Signal: DAD1B,Sig=280.0,4.0 Ref=550.0,80.0

RT [min]	Type	Width [min]	Area	Height	Area%
0.292	VV	0.30	300.36	48.10	100.00
		**Sum**	**300.36**		

Figure S7. LC-MS trace and peak area for compound **6**.

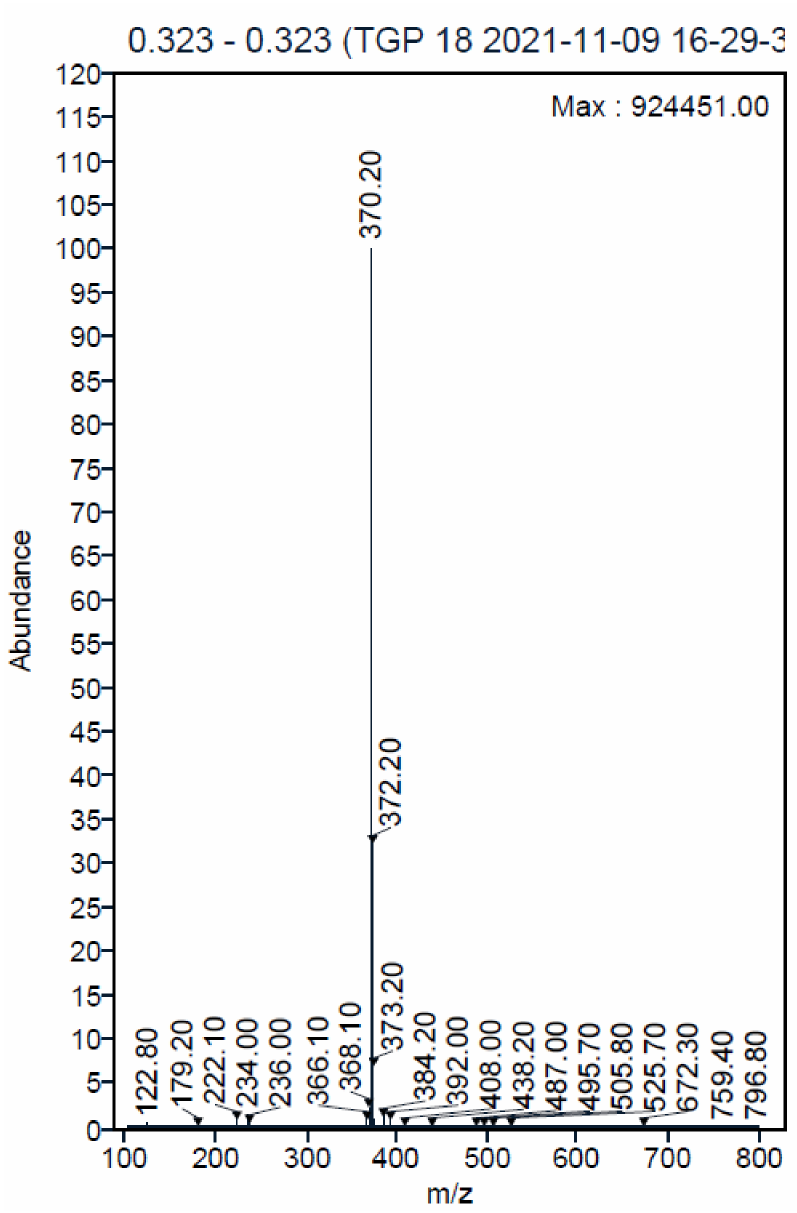

Figure S8. Mass spectrum of compound **6**.

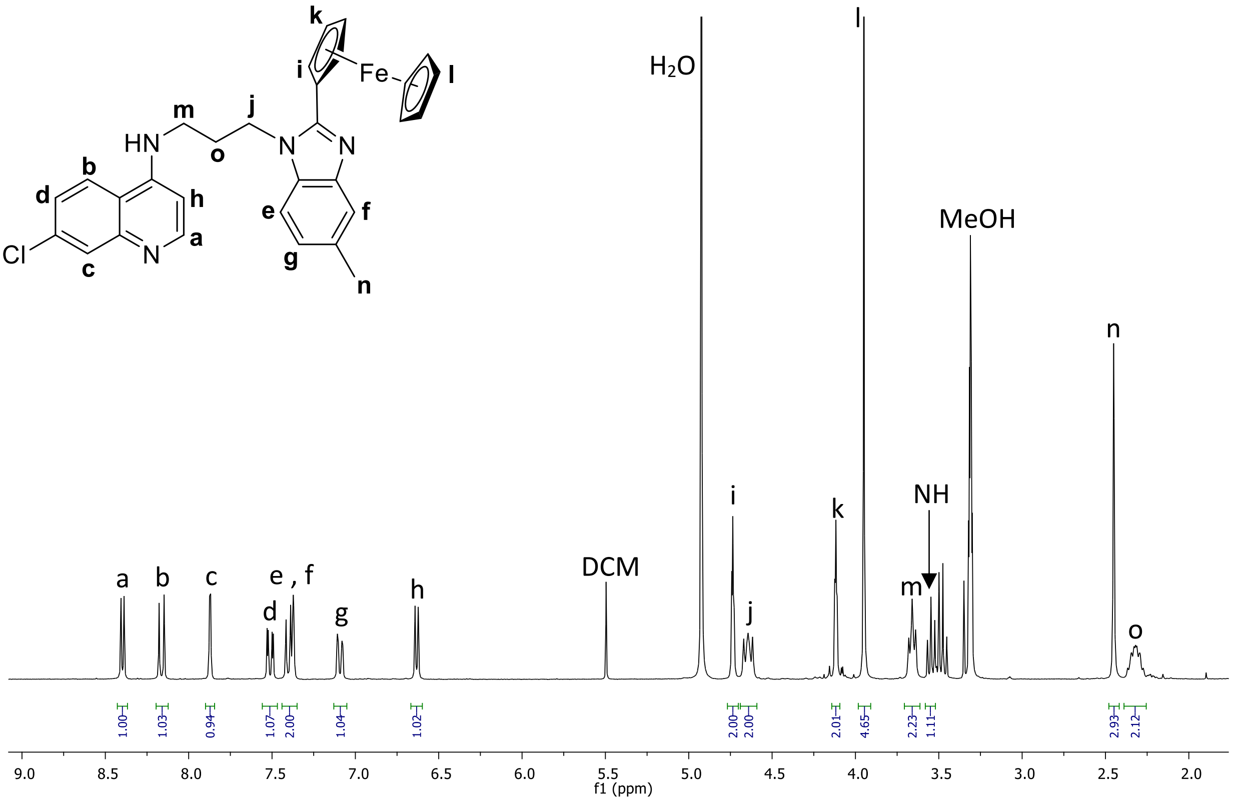

Figure S9: ^{1}H NMR spectrum of the aminoquinoline-benzimidazole ferrocenyl hybrid **C1**, in MeOD.

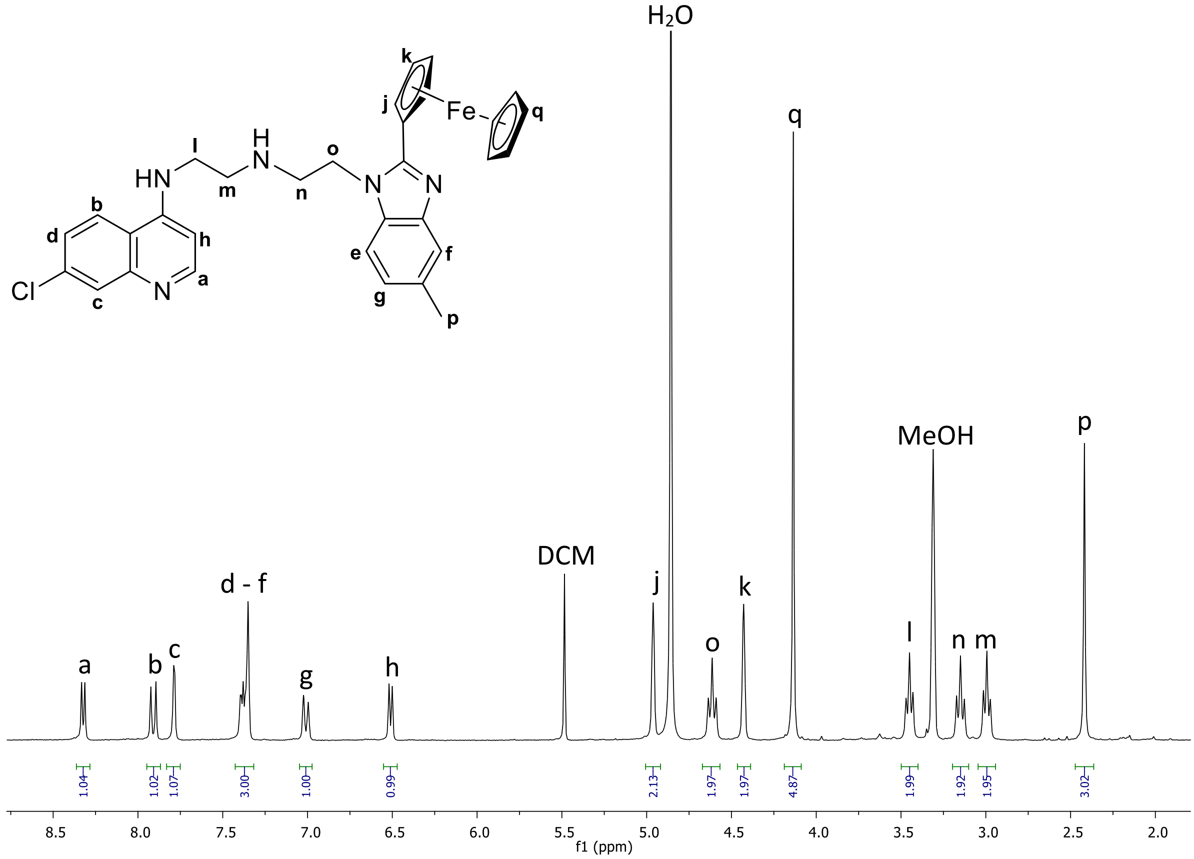

Figure S10: ^{1}H NMR spectrum of the aminoquinoline-benzimidazole ferrocenyl hybrid **C2**, in MeOD.

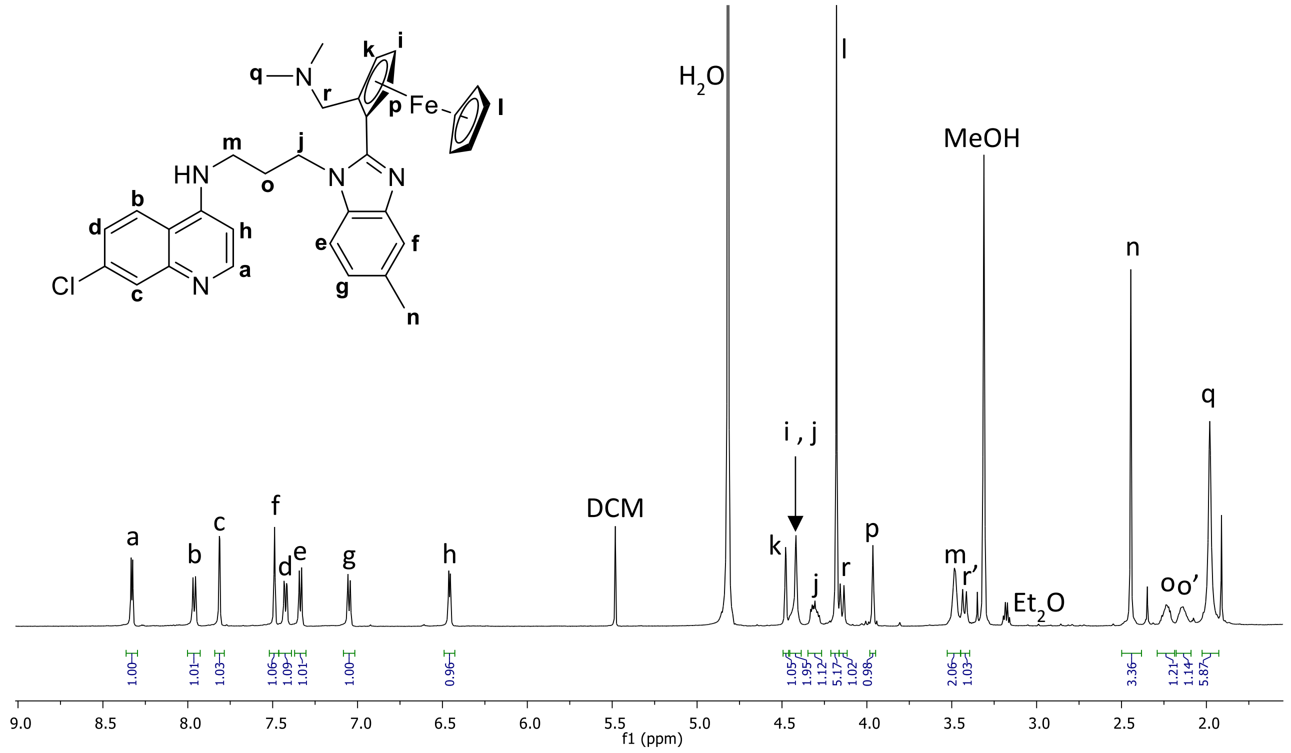

Figure S11. ^{1}H NMR spectrum of the aminoquinoline-benzimidazole ferrocenyl hybrid **C3**, in MeOD.

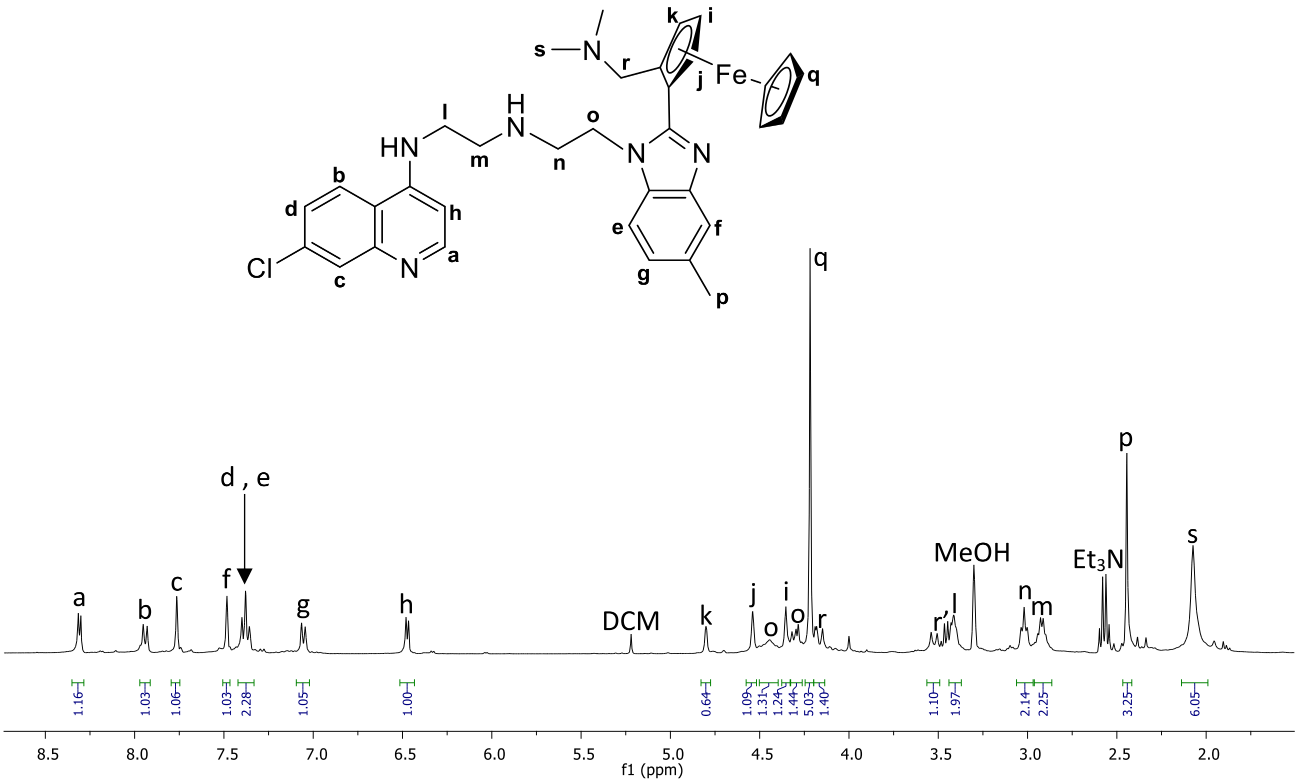

Figure S12. ^{1}H NMR spectrum of the aminoquinoline-benzimidazole ferrocenyl hybrid **C4**, in MeOD.

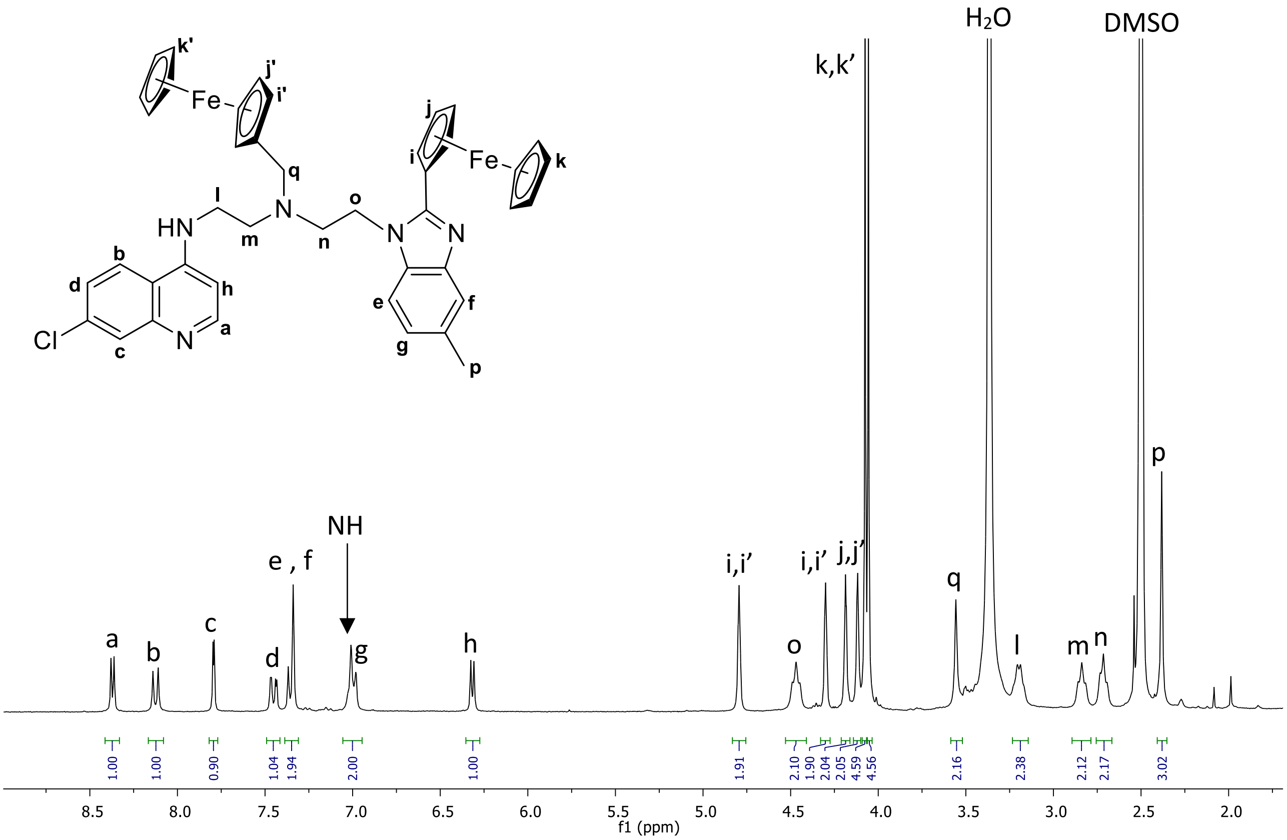

Figure S13. ^{1}H NMR spectrum of the aminoquinoline-benzimidazole ferrocenyl hybrid **C5**, in DMSO.

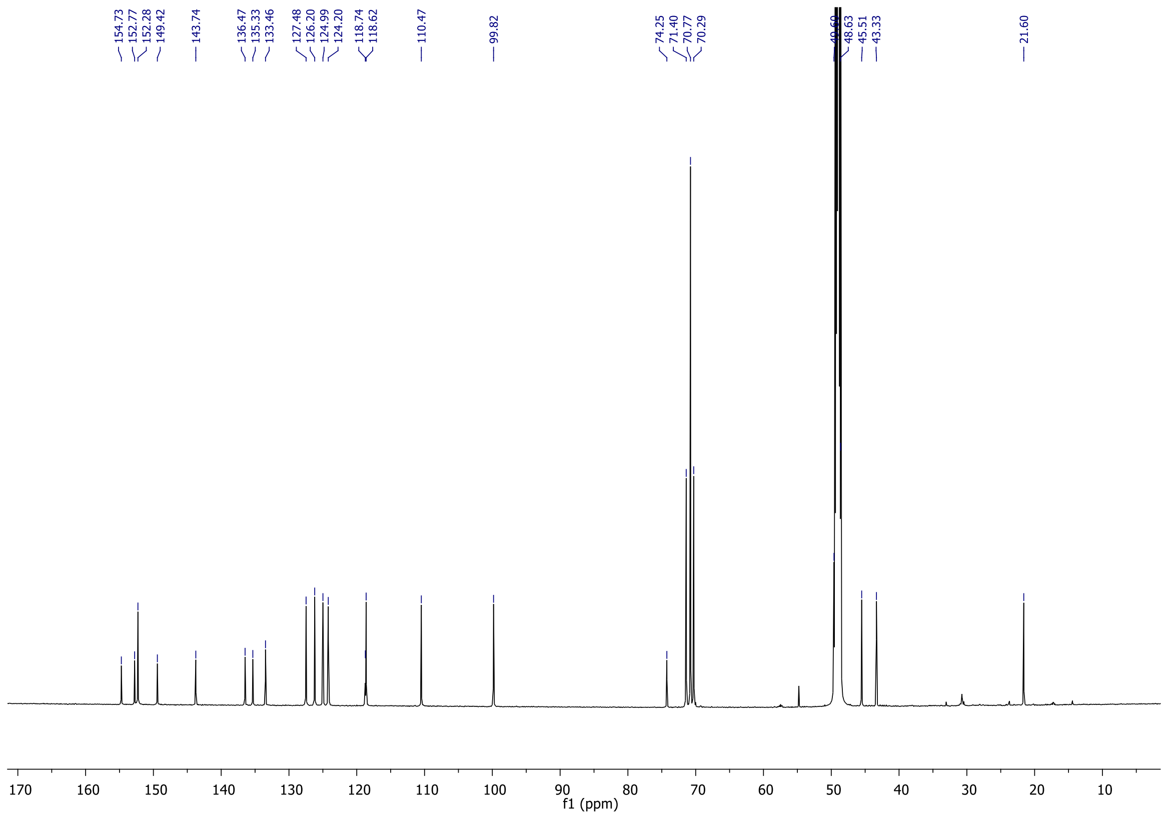

Figure S14: $^{13}C\{^{1}H\}$ NMR spectrum of the ferrocenyl hybrid **C2**, in MeOD.

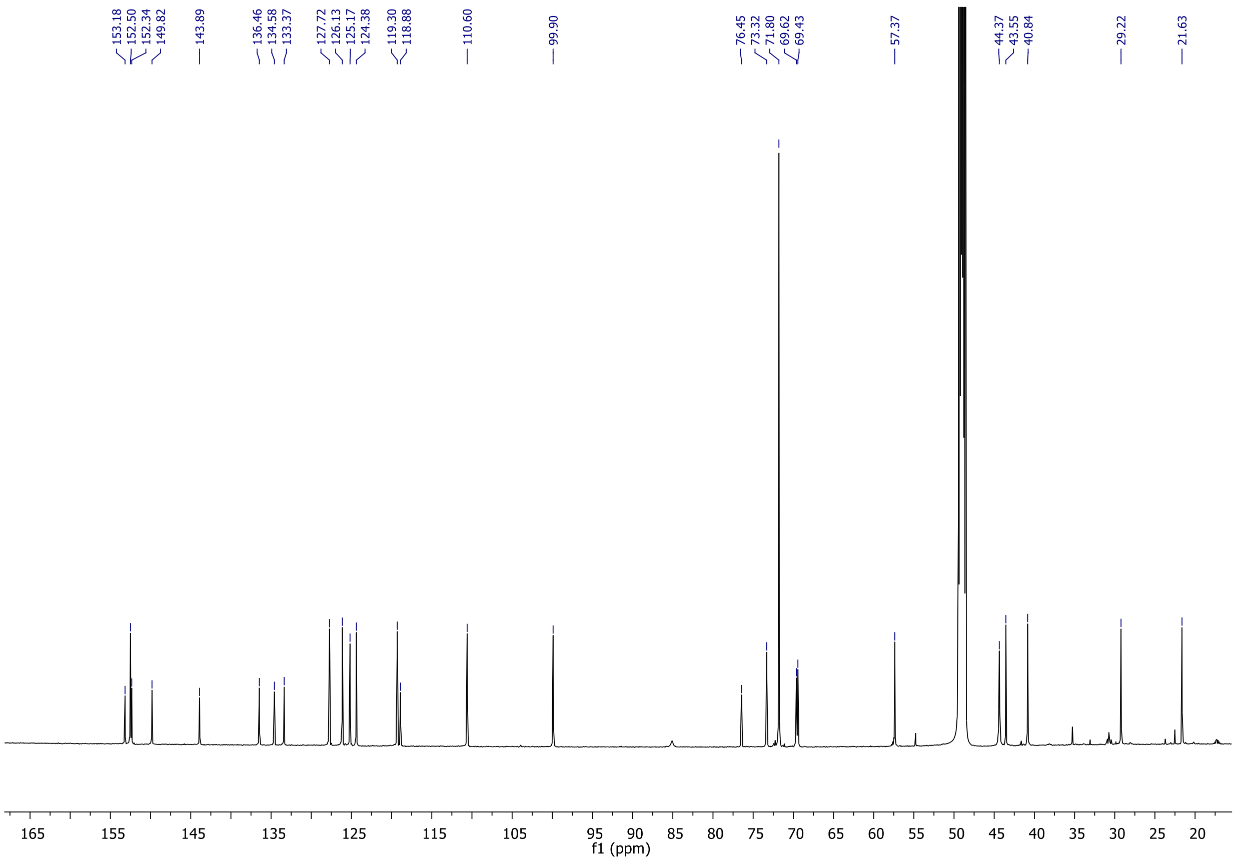

Figure S15. $^{13}C\{^{1}H\}$ NMR spectrum of the ferrocenyl hybrid **C3**, in MeOD.

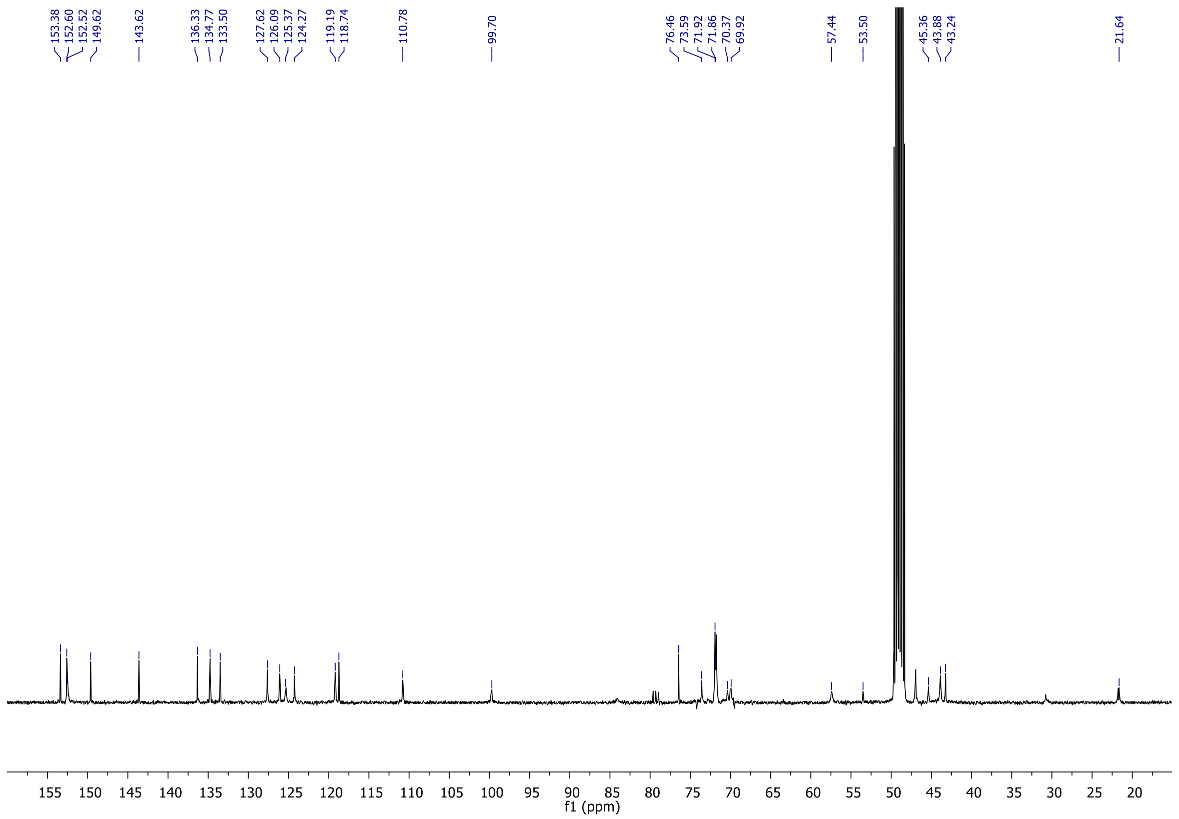

Figure S16. $^{13}C\{^1H\}$ NMR spectrum of the ferrocenyl hybrid **C4**, in MeOD.

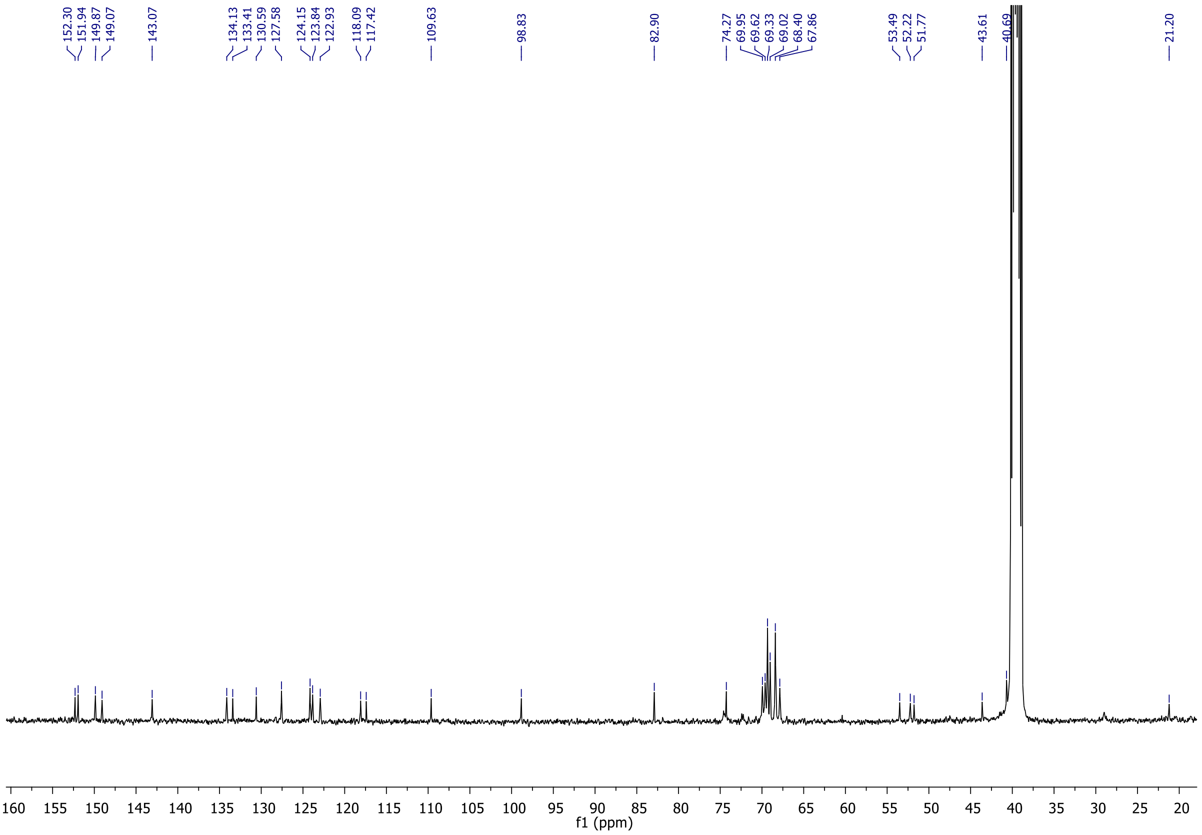

Figure S17. $^{13}C\{^1H\}$ NMR spectrum of the ferrocenyl hybrid **C5**, in DMSO.

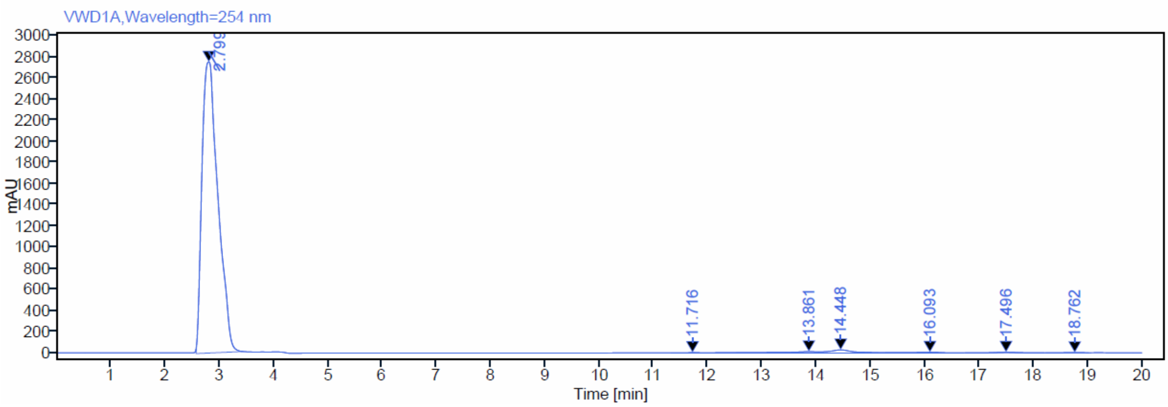

Signal: VWD1A,Wavelength=254 nm

RT [min]	Type	Width [min]	Area	Height	Area%	Name
2.799	MM m	0.30	56285.97	2751.73	96.82	
11.716	BV	0.58	35.26	2.95	0.06	
13.861	VV	2.14	536.90	11.78	0.92	
14.448	VV	1.17	833.31	27.77	1.43	
16.093	VB	1.50	246.39	5.76	0.42	
17.496	BB	1.46	145.92	5.85	0.25	
18.762	BV	0.79	51.63	2.25	0.09	
		Sum	58135.38			

Figure S18. HPLC trace of complex **C1**.

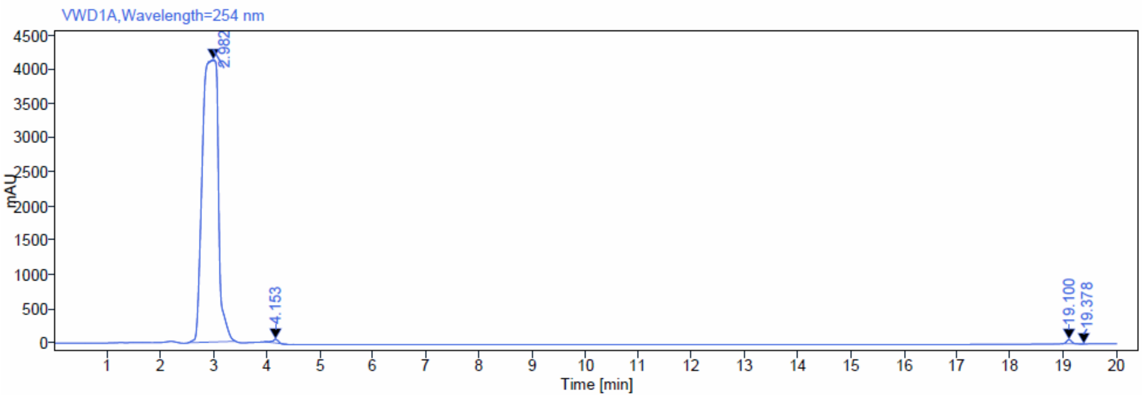

Signal: VWD1A,Wavelength=254 nm

RT [min]	Type	Width [min]	Area	Height	Area%	Name
2.982	MM m	0.24	86097.57	4137.39	99.09	
4.153	MM m	0.12	380.44	54.35	0.44	
19.100	MM m	0.10	407.67	61.76	0.47	
19.378	MM n	0.00	0.01	0.03	0.00	
		Sum	86885.69			

Figure S19. HPLC trace of complex **C2**.

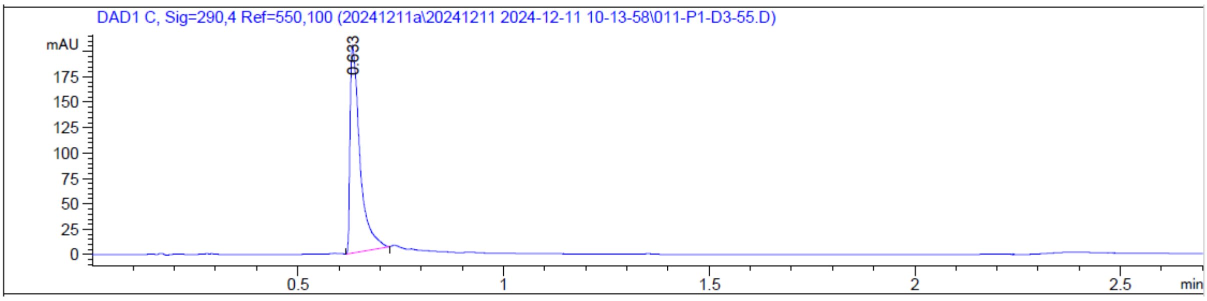

Signal 3: DAD1 C, Sig=290,4 Ref=550,100

Peak #	RetTime [min]	Type	Width [min]	Area [mAU*s]	Height [mAU]	Area %
1	0.633	BB	0.0255	339.03140	202.73659	100.0000
Totals :				339.03140	202.73659	

Figure S20. LC trace of complex **C2**.

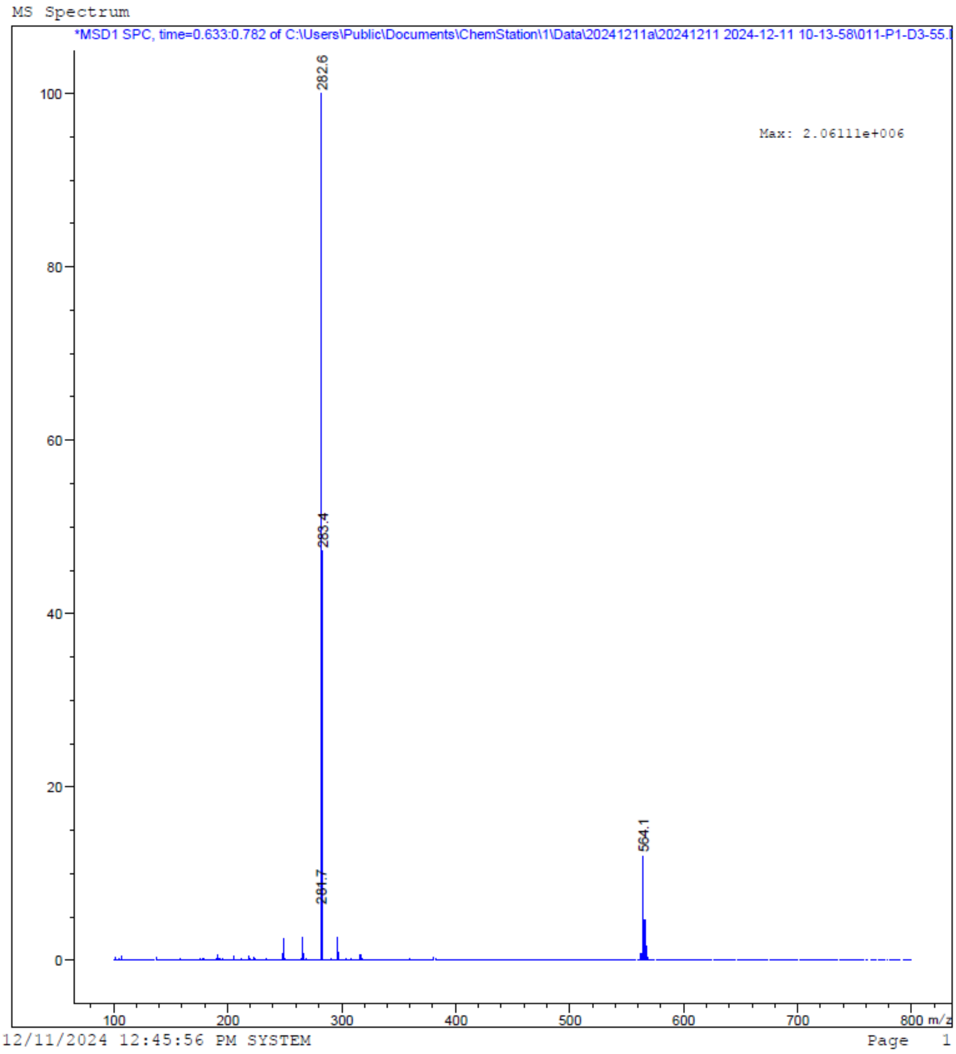

Figure S21. Mass spectrum of complex **C2**.

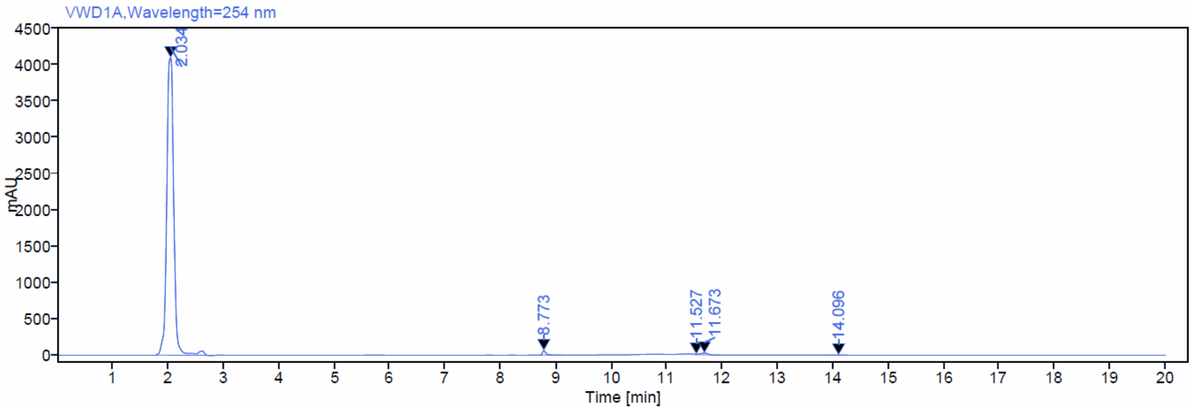

Signal: VWD1A,Wavelength=254 nm

RT [min]	Type	Width [min]	Area	Height	Area%	Name
2.034	MM m	0.12	35995.32	4101.29	98.45	
8.773	VB	0.41	279.75	63.41	0.77	
11.527	MM m	0.02	8.12	7.20	0.02	
11.673	MB m	0.14	238.98	25.53	0.65	
14.096	VB	0.56	38.92	2.59	0.11	
		Sum	36561.08			

Figure S22. HPLC trace of complex **C3**.

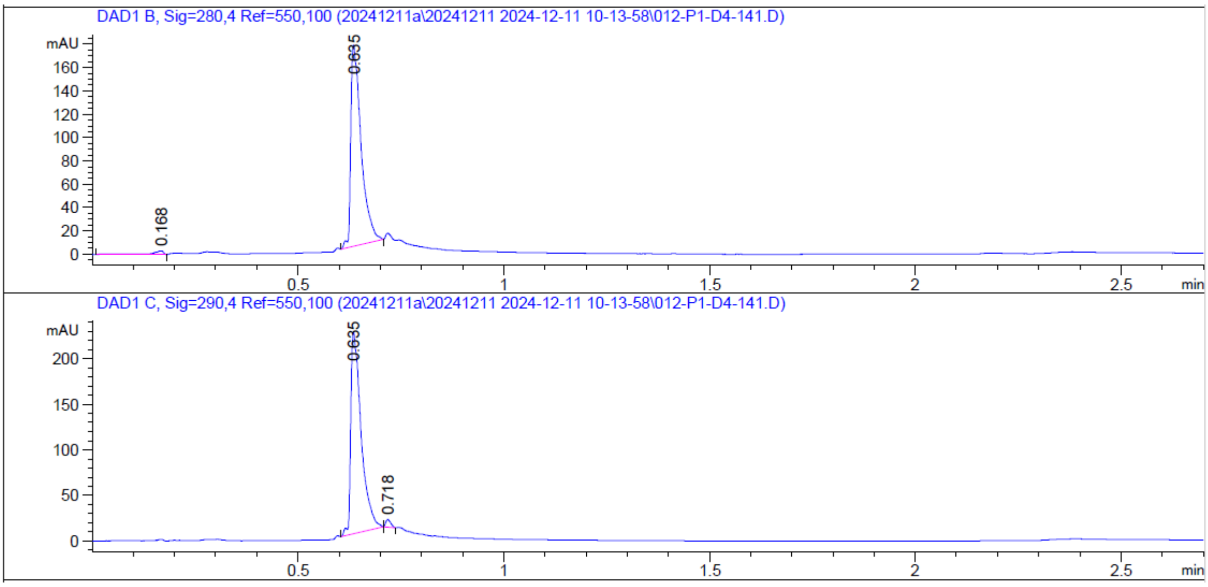

Signal 2: DAD1 B, Sig=280,4 Ref=550,100

Peak #	RetTime [min]	Type	Width [min]	Area [mAU*s]	Height [mAU]	Area %
1	0.168	BB	0.0311	7.12730	3.06806	2.3036
2	0.635	BB	0.0236	302.26465	171.45454	97.6964
Totals :				309.39195	174.52260	

Signal 3: DAD1 C, Sig=290,4 Ref=550,100

Peak #	RetTime [min]	Type	Width [min]	Area [mAU*s]	Height [mAU]	Area %
1	0.635	BB	0.0235	388.41232	220.67606	98.2606
2	0.718	BB	0.0138	6.87551	8.15200	1.7394
Totals :				395.28784	228.82806	

Figure S23. LC trace of complex **C4**.

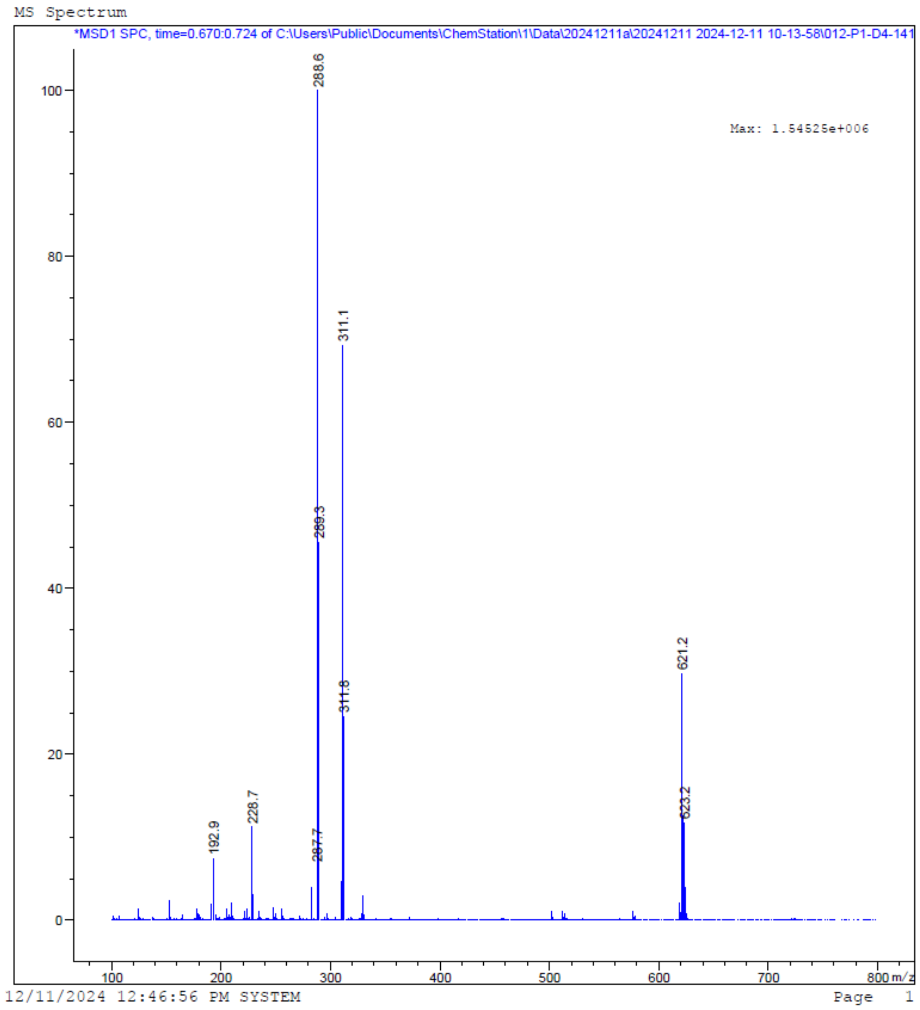

Figure S24. Mass spectrum of complex **C4**.

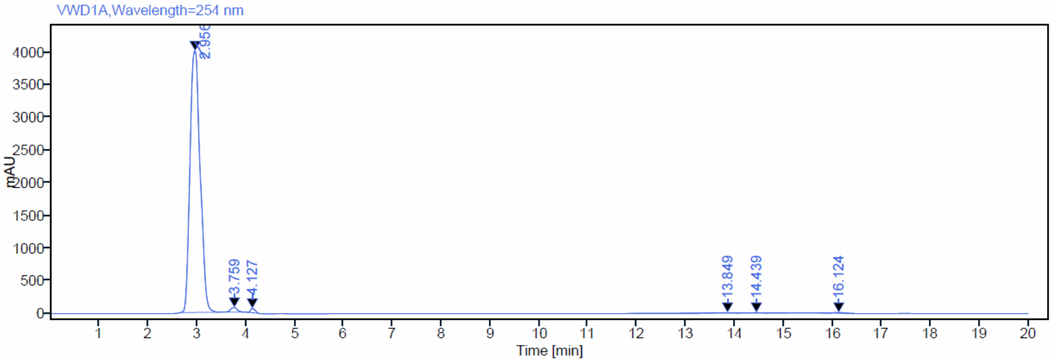

Signal: VWD1A,Wavelength=254 nm

RT [min]	Type	Width [min]	Area	Height	Area%	Name
2.956	MM m	0.24	57860.04	4001.66	96.89	
3.759	MM m	0.16	648.61	67.93	1.09	
4.127	MM m	0.12	407.89	63.40	0.68	
13.849	VV	2.13	364.71	8.46	0.61	
14.439	VB	1.02	213.99	7.40	0.36	
16.124	BB	0.75	219.42	12.25	0.37	
		Sum	59714.64			

Figure S25. HPLC trace of complex **C5**

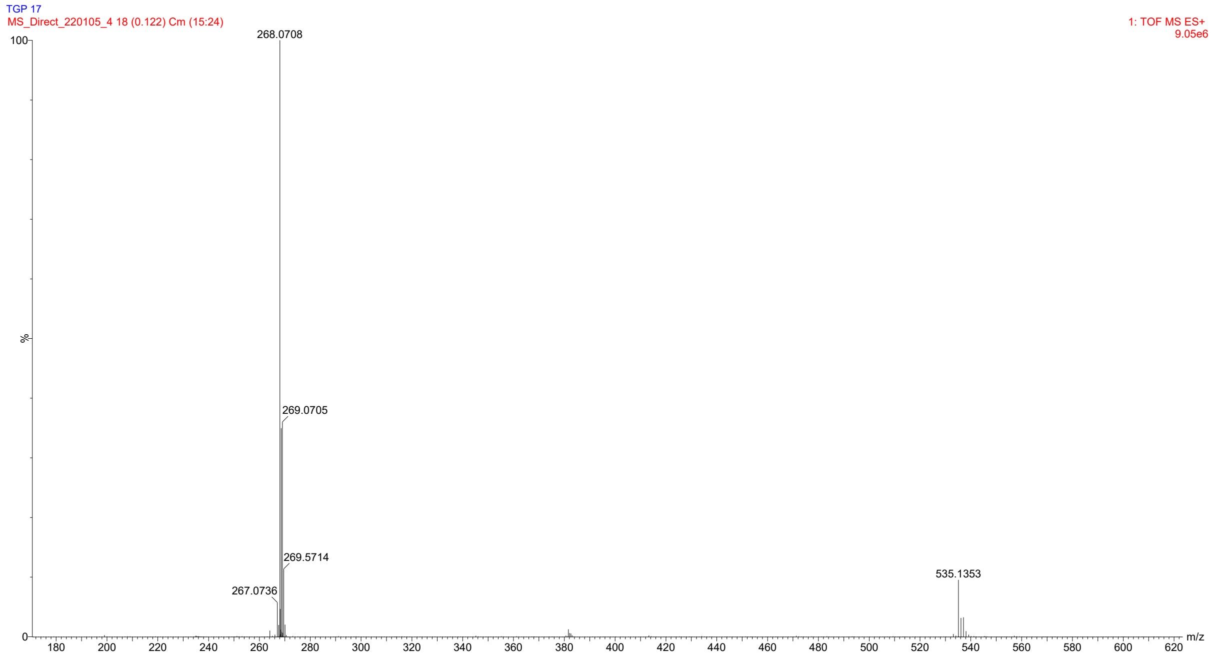

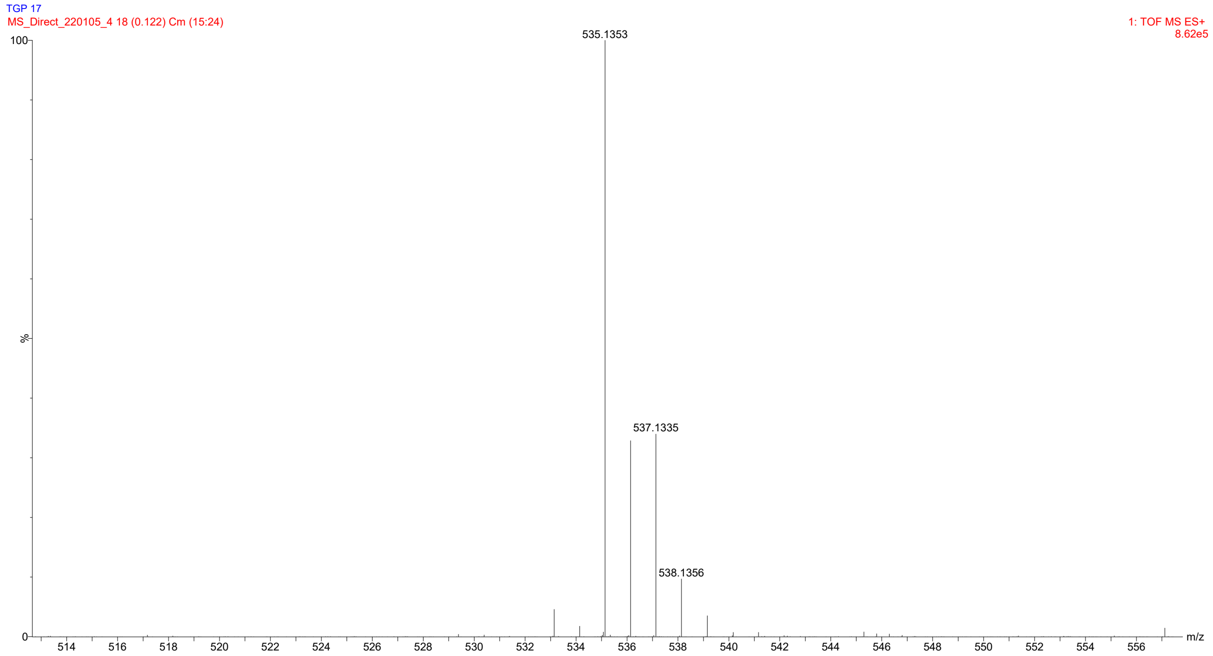

Figure S26. Mass spectra of complex **C1**.

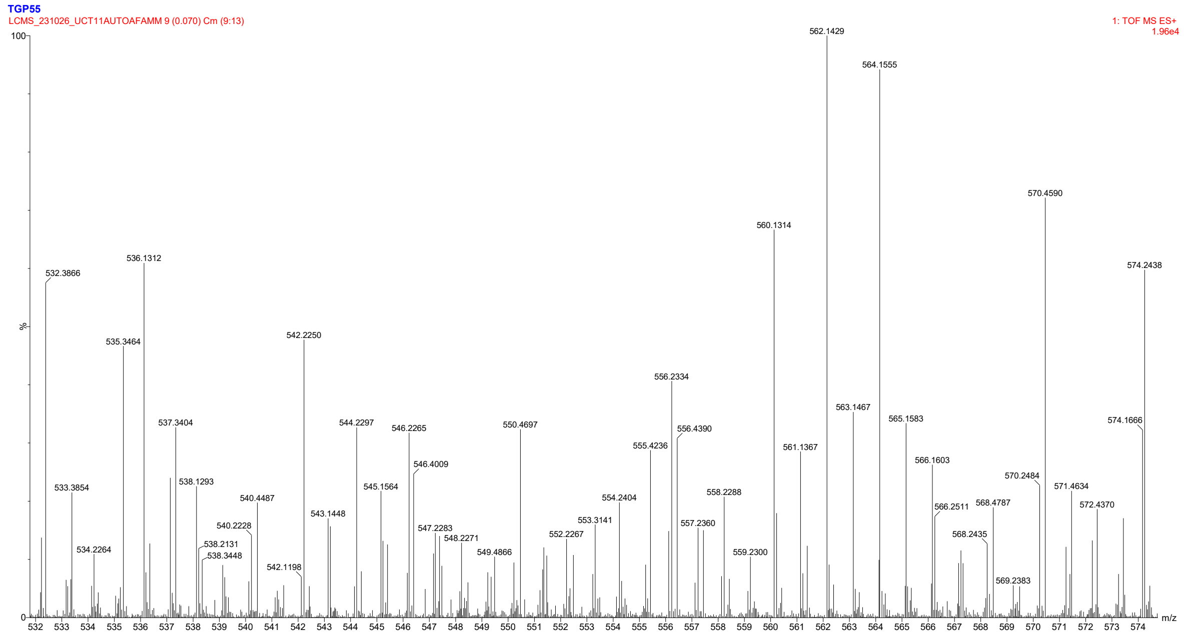

Figure S27. Mass spectrum of complex **C2**.

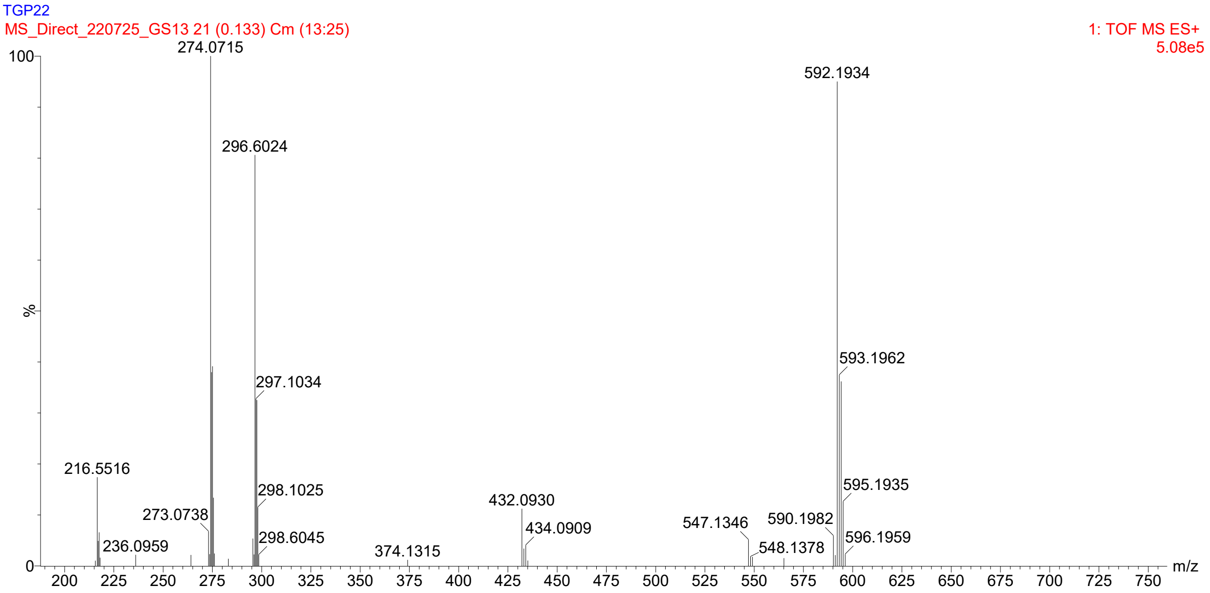

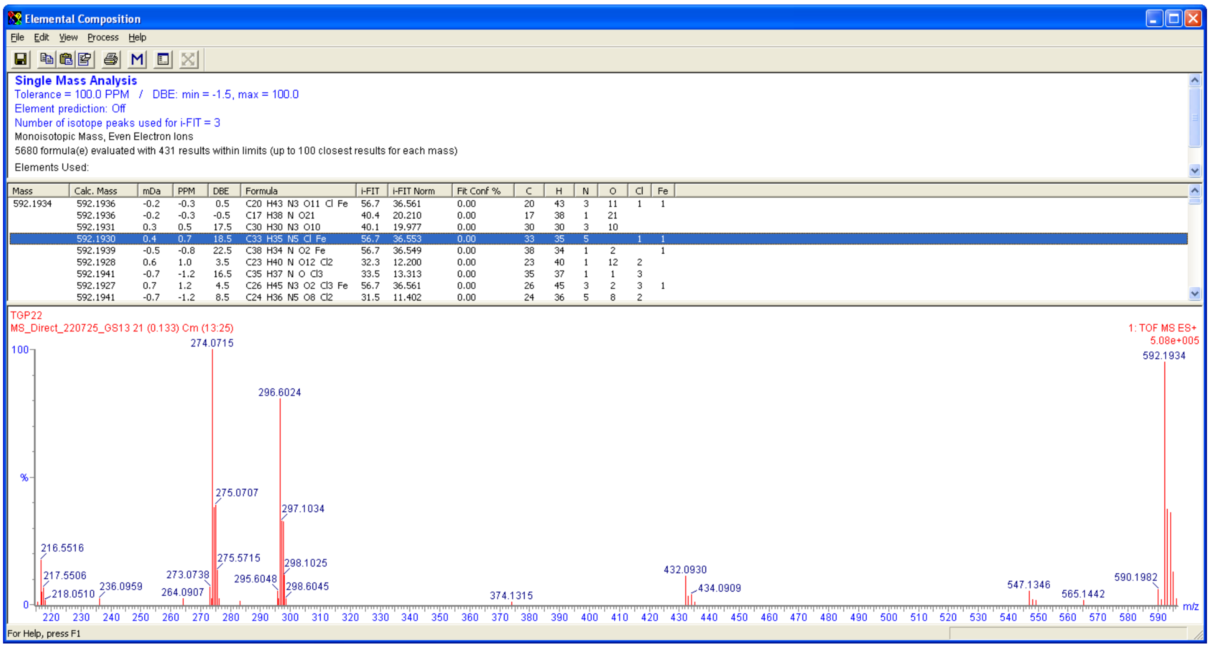

Mass	Calc. Mass	mDa	PPM	DBE	Formula	i-FIT	i-FIT Norm	Fit Conf %	C	H	N	O	Cl	Fe
592.1934	592.1936	-0.2	-0.3	0.5	C20 H43 N3 O11 Cl Fe	56.7	36.561	0.00	20	43	3	11	1	1
	592.1936	-0.2	-0.3	-0.5	C17 H38 N O21	40.4	20.210	0.00	17	38	1	21		
	592.1931	0.3	0.5	17.5	C30 H30 N3 O10	40.1	19.977	0.00	30	30	3	10		
	592.1930	0.4	0.7	18.5	C33 H35 N5 Cl Fe	56.7	36.553	0.00	33	35	5		1	1
	592.1939	-0.5	-0.8	22.5	C38 H34 N O2 Fe	56.7	36.549	0.00	38	34	1	2		1
	592.1928	0.6	1.0	3.5	C23 H40 N O12 Cl2	32.3	12.200	0.00	23	40	1	12	2	
	592.1941	-0.7	-1.2	16.5	C35 H37 N O Cl3	33.5	13.313	0.00	35	37	1	1	3	
	592.1927	0.7	1.2	4.5	C26 H45 N3 O2 Cl3 Fe	56.7	36.561	0.00	26	45	3	2	3	1
	592.1941	-0.7	-1.2	8.5	C24 H36 N5 O8 Cl2	31.5	11.402	0.00	24	36	5	8	2	

Figure S28. Mass spectrum and elemental composition of complex **C3**.

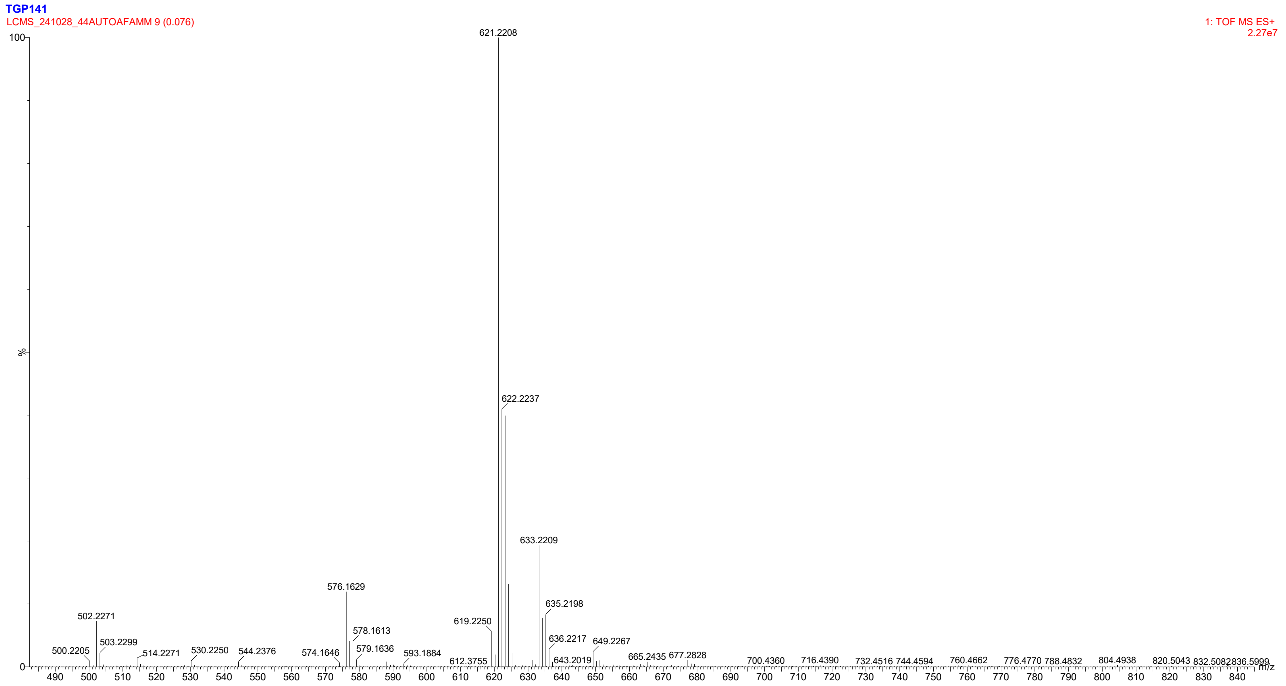

Figure S29. Mass spectrum of complex **C4**.

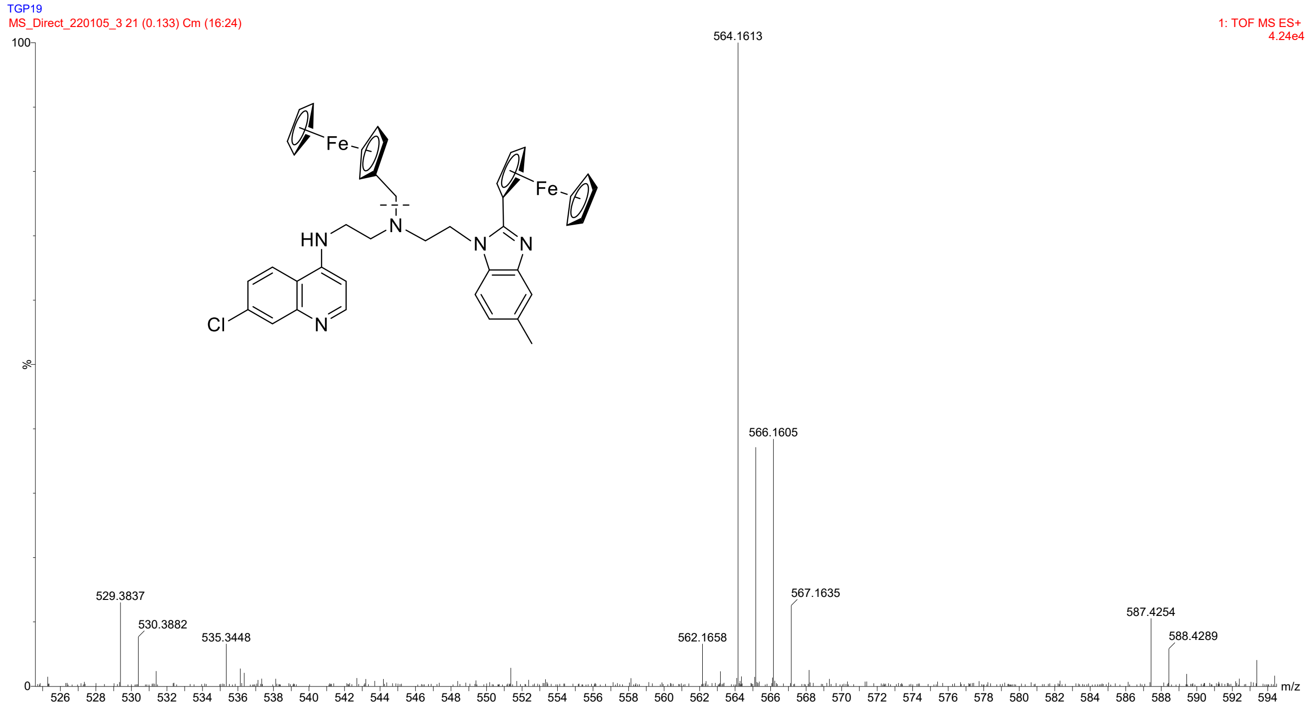

Figure S30. Mass spectrum of complex **C5**, with the structure identifying the predicted cleavage site.

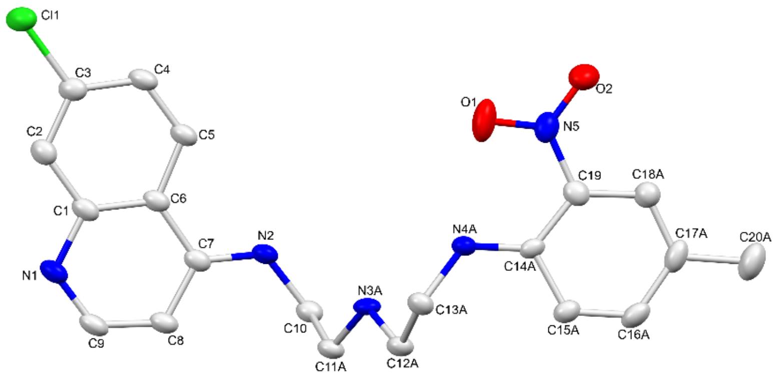

Part of the diethylenetriamine linker, the substituted phenyl ring, and the methyl group, are disordered, with refined site occupancy factors of 0.698(4) for part A and 0.302(4) for part B.

Figure S31. Molecular structure of the nitro-containing quinoline precursor, **4**, showing one of the disordered moieties and one methanol solvent molecule. Hydrogen atoms have been omitted for clarity. Ellipsoids are shown at 30% probability level.

Table S1. Crystallographic data and refinement parameters for precursor **4** · CH_3OH.

**Formula Unit**	$C_{20}H_{22}ClN_5O_2 \cdot CH_4O$	**F(000)**	456
**Formula Weight**	431.92	**Crystal Size (mm)**	0.19 x 0.26 x 0.33
**Crystal System**	Triclinic	**Temperature (K)**	100
**Space Group**	P-1	**Scan Range (°)**	1.9 < θ < 28.4
**a, b, c (Å)**	9.3161(3), 10.5846(3), 11.5750(3)	**Unique Reflections**	5168
**α, β, γ (°)**	109.289(1), 99.401(1), 99.098(1)	**R_{int}**	0.035
**Volume (Å^3)**	1034.91(5)	**Observed Data [I>2σ(I)]**	4466
**Z**	2	**R, wR2**	0.0367, 0.0953
**Density$_{calc}$ (g/cm^3)**	1.386	**Goodness-of-fit**	1.04
**μ (mm^{-1})**	0.219	**Min, Max Δρ (e.Å^{-3})**	-0.68, 0.72

Table S2. Crystallographic data and refinement parameters for complex **C1** · 2CH_3OH.

**Formula Unit**	$C_{30}H_{27}ClFeN_4 \cdot 2(CH_4O)$	**F(000)**	628
**Formula Weight**	598.94	**Crystal Size (mm)**	0.06 x 0.16 x 0.34
**Crystal System**	Triclinic	**Temperature (K)**	173
**Space Group**	P-1	**Scan Range (°)**	1.7 < θ < 28.3
**a, b, c (Å)**	9.9121(7), 12.2506(9), 13.7562(9)	**Unique Reflections**	7198
**α, β, γ (°)**	112.040(2), 108.171(1), 92.203(1)	**R_{int}**	0.051
**Volume (Å^3)**	1448.02(18)	**Observed Data [I>2σ(I)]**	6156
**Z**	2	**R, wR2**	0.0328, 0.0844
**Density$_{calc}$ (g/cm^3)**	1.374	**Goodness-of-fit**	1.04
**μ (mm^{-1})**	0.649	**Min, Max Δρ (e.Å^{-3})**	-0.30, 0.66

Table S3. Selected bond lengths (Å) and angles (°) for complex **C1** · 2CH_3OH.

**Bond lengths (Å)**		**Torsion angle (°)**	
**$C_{20} – C_{21}$**	1.460(2)	**$C_{25} – C_{21} – C_{20} – N_2$**	27.1(3)
**$C_1 – N_1$**	1.387(2)		
**$C_6 – N_2$**	1.388(2)		

Table S4. Crystallographic data and refinement parameters for complex **C5**.

**Formula Unit**	$C_{42}H_{40}ClFe_2N_5$	**F(000)**	1584
**Formula Weight**	761.94	**Crystal Size (mm)**	0.05 x 0.10 x 0.11
**Crystal System**	Monoclinic	**Temperature (K)**	100
**Space Group**	P21/c	**Scan Range (°)**	2.1 < θ < 28.4
**a, b, c (Å)**	9.9064(9), 29.418(2), 12.1871(11)	**Unique Reflections**	8680
**α, β, γ (°)**	90, 101.177(3), 90	**R_{int}**	0.061
**Volume (Å^3)**	3484.3(5)	**Observed Data [I>2σ(I)]**	7199
**Z**	4	**R, wR2**	0.0333, 0.0778
**Density$_{calc}$ (g/cm^3)**	1.452	**Goodness-of-fit**	1.04
**μ (mm^{-1})**	0.949	**Min, Max Δρ (e.Å^{-3})**	-0.35, 0.38

Table S5. Selected bond lengths (Å) and angles (°) for complex **C5**.

**Bond lengths (Å)**		**Torsion angle (°)**	
**$C_{21} – C_{22}$**	1.462(3)	**$N_5 – C_{21} – C_{22} – C_{23}$**	11.9(3)
**$C_{32} – C_{33}$**	1.496(2)		
**$N_3 – C_{11}$**	1.468(2)		
**$N_3 – C_{12}$**	1.461(2)		
**$N_3 – C_{32}$**	1.474(2)		

Table S6. Anodic (E_{pa}) and cathodic (E_{pc}) peak potentials[a], anodic and cathodic current ratios (I_{pc}/I_{pa}), and half-wave potentials ($E_{1/2}$)[a] for ferroquine and complexes **C1** - **C5**.

**Compound**	**E_{pa} (V)**	**E_{pc} (V)**	**ΔE (V)**	**I_{pc}/I_{pa}**	**$E_{1/2}$ (V)**
**Ferrocene**	0.543	0.372	0.171	1.05	0.458
**Ferroquine**	0.613	0.488	0.125	1.00	0.550
**C1**	0.638	0.510	0.127	1.04	0.574
**C2**	0.651	0.518	0.133	1.03	0.584
**C3**	0.678	N/A[b]	N/A[b]	N/A[b]	N/A[b]
**C4**	0.662	N/A[b]	N/A[b]	N/A[b]	N/A[b]
**C5**	0.632	0.442	0.190	1.04	0.537

[a] Potential vs the Ag/AgCl reference electrode, with a scan rate (v) of 100mV.s^{-1}.

[b] Potentials not observed due to irreversible oxidation.

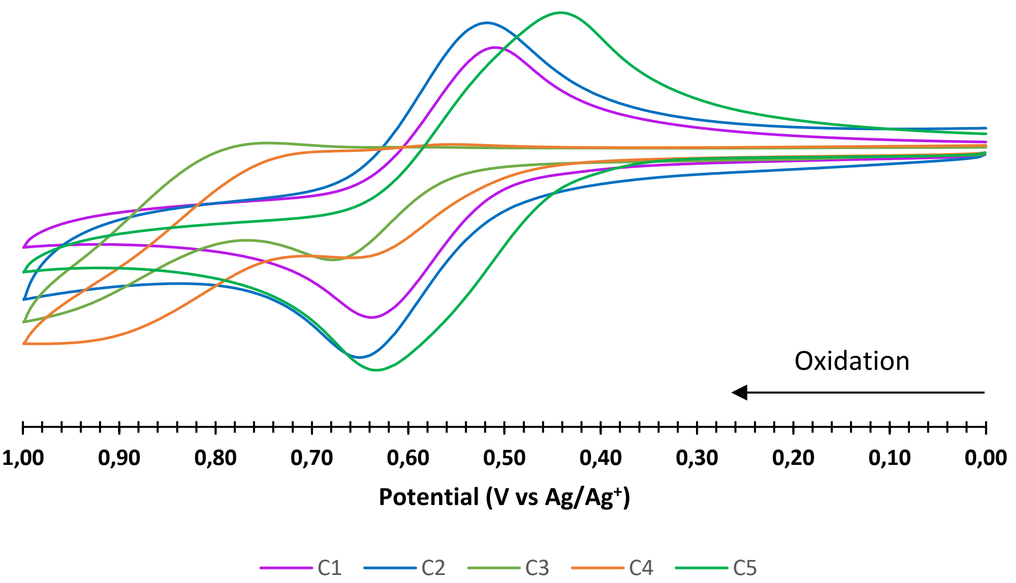

Figure S32. Cyclic voltammograms of complexes **C1** – **C5** in CH_2Cl_2.

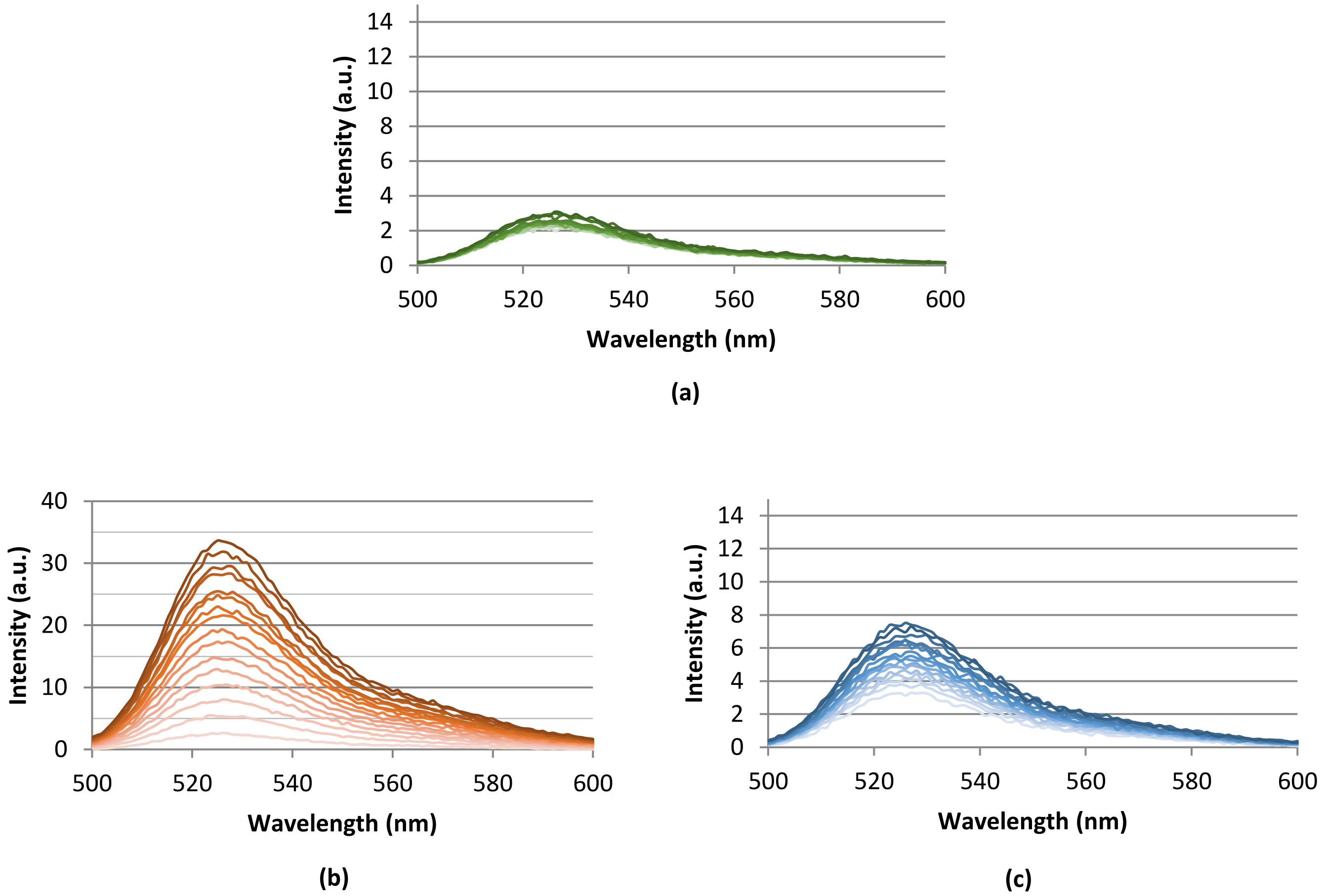

Figure S33. Changes in the fluorescence emission spectra of (a) H_2O_2 (negative control), (b) ferrocene (positive control), and (c) ferroquine, in 1:4 DMSO/PBS, following the addition of H_2O_2 and the non-fluorescent fluorescein analogue, H_2DCF, monitored over 30 minutes.

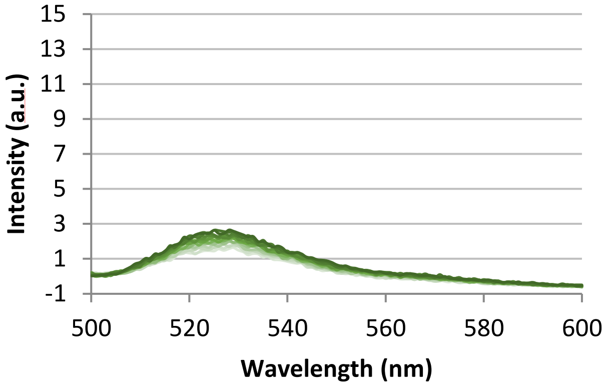

(a)

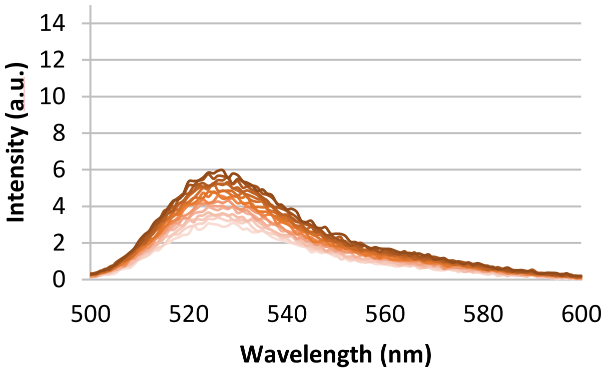

(b)

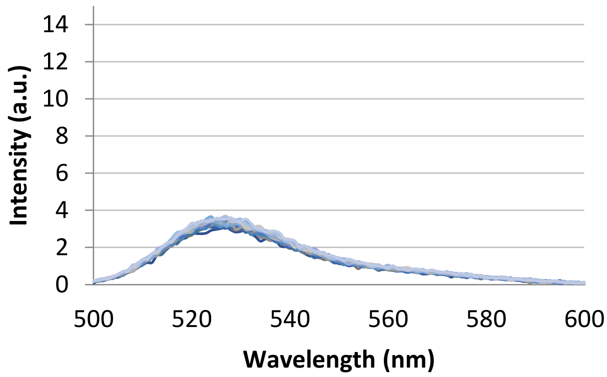

(c)

Figure S34. Changes in the fluorescence emission spectra of complex (a) **C1**, (b) **C3**, and (c) **C5**, in 1:4 DMSO/PBS, following the addition of H_2O_2 and the non-fluorescent fluorescein analogue, H_2DCF, monitored over 30 minutes.

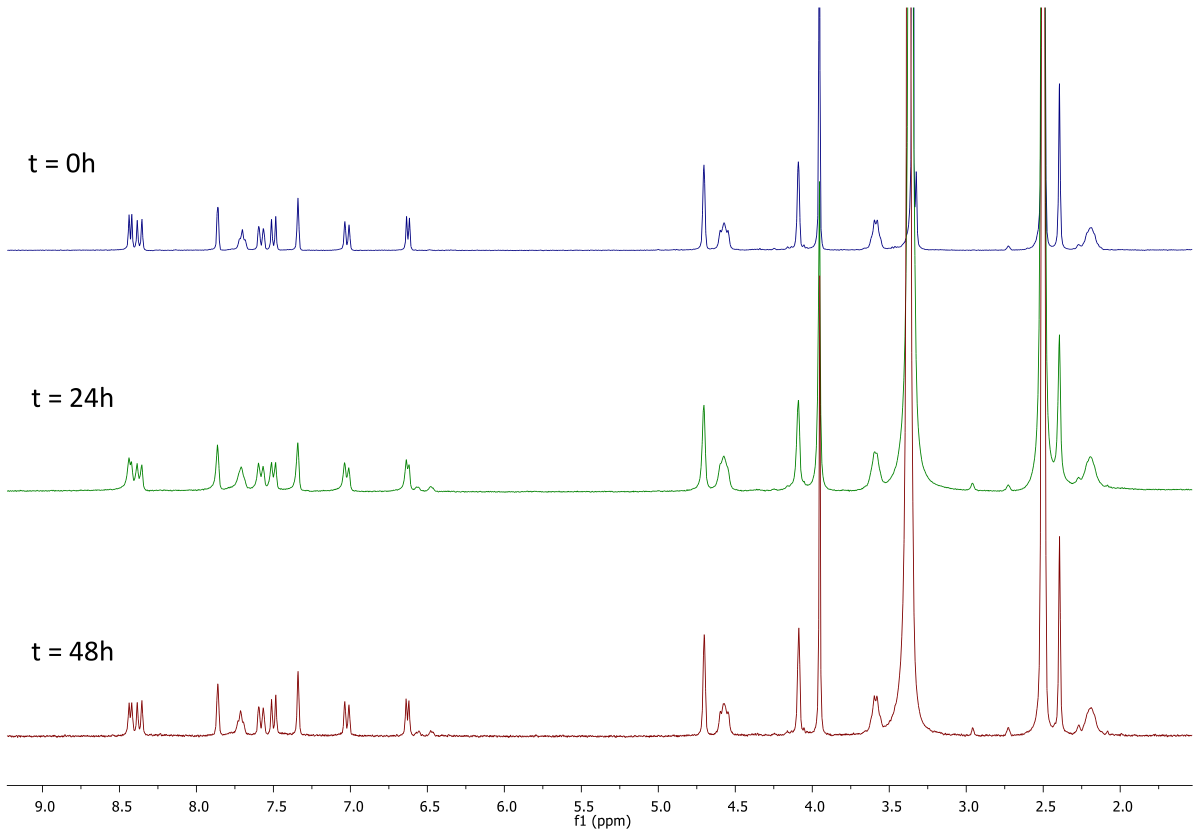

Figure S35. Stability of **C1**, in DMSO, as monitored by ^{1}H NMR spectroscopy, over 48 hours.

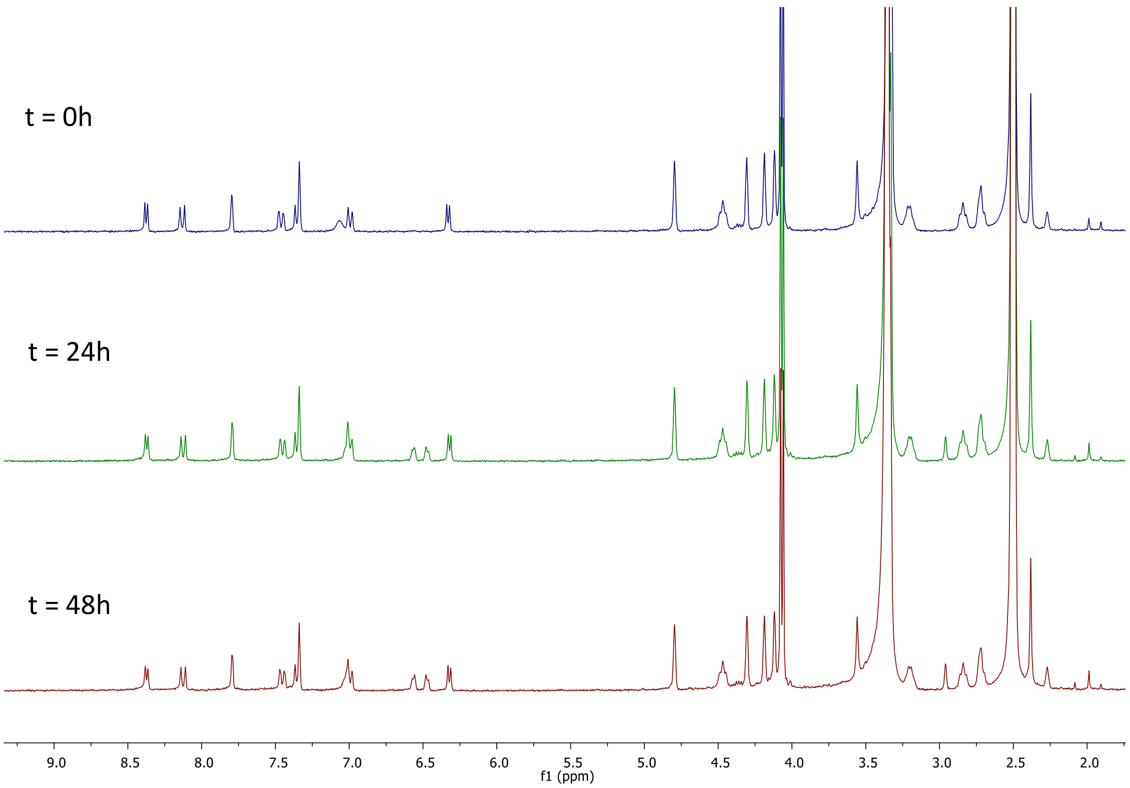

Figure S36. Stability of **C5**, in DMSO, as monitored by ^{1}H NMR spectroscopy, over 48 hours.
